# Behind the smile: forensic odontology knowledge, attitudes, and practices among dental students, interns, and practitioners in the Asir region, Saudi Arabia: a cross-sectional study

**DOI:** 10.1186/s12903-026-09465-2

**Published:** 2026-07-31

**Authors:** Hassan Ahmed Assiri, Abdullah H. Alshehri, Rema A. Alshehry, Hajer Saeed Al-serhani, Rami Abu-dahmah, Khalid Salem Alqahtani, Zayed Assiri, Mohammed Alalmai, Ali Azhar Dawasaz

**Affiliations:** 1 Department of Diagnostic Sciences and Oral Biology and Periodontology, College of Dentistry, King Khalid University, Abha, 61421 Saudi Arabia; 2 Department of Prosthodontics, College of Dentistry, King Khalid University, Abha, 61421 Saudi Arabia; 3 Internship Program, College of Dentistry, King Khalid University, Abha, 61421 Saudi Arabia; 4 General dental practice, Kanaf Dental Center, Abha, Saudi Arabia; 5 Department of Oral and Maxillofacial Surgery, Asir Central Hospital, Abha, Saudi Arabia

**Keywords:** Forensic odontology, Forensic dentistry, Dental records, Knowledge attitudes practices, Dental education, Saudi Arabia

## Abstract

**Background:**

Forensic odontology contributes to human identification, age estimation, and medico-legal documentation, yet dental personnel’s preparedness in many regions is unclear. This study assessed the knowledge, attitudes, and practices (KAP) regarding forensic odontology and dental record-keeping among dental students, interns, and practitioners in the Aseer region, Saudi Arabia, and examined the associations among the three domains.

**Methods:**

A cross-sectional survey was conducted among 73 dental personnel across multiple institutions in Abha and Khamis Mushait. Participants were recruited by convenience sampling; the study was designed as a preliminary, single-region assessment. A structured questionnaire assessed knowledge (six single-best-answer items), attitudes (four pro-field Likert items), and record-keeping practices (three adequacy items). Knowledge was categorised using Bloom’s cut-offs. Non-parametric tests (Mann–Whitney, Kruskal–Wallis) and Spearman correlations were used; internal consistency was assessed by Cronbach’s α.

**Results:**

Overall knowledge was low (mean 40.6%; 50.7% poor, 9.6% good), with the weakest performance on record-retention standards (19.2% correct), cheiloscopy (23.3%), and adult age estimation (23.3%). Attitudes were consistently favourable (mean 14.8/20; α = 0.93) and did not differ across subgroups. Record-keeping practices were moderate (mean 5.7/9); only 19.2% reported appropriate record retention, and 43.8% had encountered a request for dental records, which were incomplete or unavailable in 17.8% of the sample. Knowledge correlated positively with attitudes (ρ = 0.45, *p* = 0.0004) and practices (ρ = 0.26, *p* = 0.042), whereas attitudes and practices were unrelated (ρ = 0.13, *p* = 0.33). Almost half of respondents (46.6%) reported no undergraduate forensic-odontology training.

**Conclusions:**

In this preliminary single-region study, dental personnel in the Aseer region held favourable attitudes but demonstrate limited forensic-odontology knowledge and inconsistent record-keeping, with knowledge—not attitude—linked to practice. Integrating forensic odontology into dental curricula and continuing education, alongside standardised record-keeping systems, is needed to strengthen regional medico-legal and disaster-victim-identification preparedness.

**Supplementary Information:**

The online version contains supplementary material available at https://doi.org/10.1186/s12903-026-09465-2.

## Introduction

Forensic odontology is a branch of dentistry that applies dental science and expertise for legal purposes, including civil and criminal matters [1]. It operates on the premise that teeth and their associated structures are highly resilient and generally survive conditions such as fire, trauma, and advanced decomposition longer than most other parts of the body. Owing to this durability, dental evidence has become a significant factor in identifying individuals who cannot be recognized by sight or fingerprints [1, 2]. Antemortem records include, but are not restricted to, radiographs, clinical notes, treatment histories, odontograms, photographs, and prosthetic records [3]. Even where a forensic odontologist is available, accurate postmortem identification is impossible without adequate antemortem data [3]. In a study of dental practitioners in India, Astekar et al. found that only 38% of dentists maintained dental records, while 62% reported keeping none at all [3]. The deficiencies identified included missing radiographic details, incomplete dentition records, and failure to document prescribed medications [3]. Saudi Arabia presents a particularly compelling context for forensic odontology. The Kingdom hosts the annual Hajj, one of the largest recurrent mass gatherings in the world [4]. Such gatherings carry a recognized risk of mass-casualty events requiring rapid disaster victim identification, and this challenge is compounded when victims lack conventional identification. In parallel, road traffic crashes remain a major cause of mortality in the Kingdom; Saudi Arabia recorded one of the highest road-fatality rates among high-income countries in 2016, and although national figures have since declined, the Aseer region has been reported among those showing persistent trends [5]. Several studies conducted across different regions of Saudi Arabia have examined dental practitioners’ and students’ familiarity with forensic odontology [6–8]. Collectively, these reports indicate consistently low levels of knowledge and limited engagement with forensic principles among both groups. For instance, Abdul et al. found that only 40.9% of undergraduate dental students in Riyadh recognized teeth as a source of DNA, compared with 75% of postgraduates [6]. Such findings underscore the need to introduce forensic odontology earlier in the dental curriculum and to strengthen continuing professional development for practicing clinicians so that the medico-legal importance of the discipline is more widely recognized. Despite the growing significance of forensic odontology in disaster victim identification (DVI), legal investigations, and human identification, no published study has specifically evaluated the knowledge, attitudes, and practices (KAP) of dental practitioners regarding forensic odontology in the Aseer region of Saudi Arabia—particularly among clinicians at King Khalid University Dental Hospital, Aseer Central Hospital, and the Armed Forces Hospitals Southern Region (AFHSR), Khamis Mushait. To the best of our knowledge, this is the first such study in the region. The present study therefore aims to assess the level of knowledge of forensic odontology among dental practitioners in the Aseer region; to determine their attitudes toward the significance and application of forensic dentistry in routine practice; to examine current practices related to dental record maintenance and forensic documentation; to identify barriers limiting the application of forensic odontology principles in clinical settings; and to examine correlations among knowledge, attitude, and practice levels.

## Materials and methods

### Study design and setting

We conducted a cross-sectional, questionnaire-based study in the Aseer region of southwestern Saudi Arabia over March-April 2026. The participants were included from King Khalid University, College of Dentistry, and the other private and government sectors in the region. This was a preliminary, single-region study based on a convenience sample. Given the modest sample size and non-probability sampling, the findings are intended to be exploratory and hypothesis-generating rather than statistically definitive or generalizable to the wider Saudi dental workforce; subgroup comparisons involving small strata should be interpreted with particular caution.

### Study population and sampling

Participants were recruited through convenience sampling. We considered eligible any dentist, postgraduate resident, intern, or clinical-year undergraduate (fifth or sixth year) who was practicing or training in the Aseer region at the time of the survey. Those working elsewhere, and questionnaires returned incomplete, were not included. This yielded 73 complete responses, all of which entered the analysis.

### Questionnaire development and content

The questionnaire was self-administered and written in English. In designing it, we drew on existing KAP instruments used in Saudi and neighboring populations [6–8] and adapted them to the clinical realities and medico-legal framework of the region. The final instrument ran to 30 items grouped into six sections. The first gathered demographic and professional details — age, gender, qualification, specialty, years of experience, primary work city, and practice setting. The second asked about prior exposure to the field, covering undergraduate teaching, any training taken since graduation, and the respondent’s own rating of their knowledge. The third section tested knowledge directly through eight items spanning the recognized applications of forensic odontology, the evidentiary weight of dental records, dental DNA, age-estimation techniques, the oral signs of child abuse, record-retention standards, cheiloscopy, and awareness of emerging artificial-intelligence tools. The fourth probed attitudes through five statements answered on a five-point Likert scale, from “strongly disagree” to “strongly agree.” The fifth turned to everyday practice, with five items on history documentation, baseline radiographs, record retention, previous medico-legal requests for records, and the barriers practitioners perceive. A final section posed two open-ended questions on training needs and on the medico-legal role of dentists in the region.

To establish content validity, the items were reviewed by 3 specialists in oral and maxillofacial radiology, who judged each one for relevance and clarity. Formal pilot testing, construct-validity testing, and test–retest reliability were not performed; this is acknowledged as a limitation. The complete questionnaire is provided as Supplementary Material.

### Scoring of the KAP domains

Only the knowledge items with an unambiguous correct answer were scored: a correct response earned one point, while incorrect, “do not know,” and blank responses earned none. Of the eight knowledge-related items, the six single-best-answer items (each with one objectively correct response) were scored to yield the 0–6 knowledge score reported in Table 2; the multiple-response “recognized applications” item and the “awareness of artificial intelligence” item were analysed descriptively and were not scored. We expressed each respondent’s total as a percentage and graded it as poor (< 50%; i.e., 0–2 of 6 items correct), moderate (50–75%; 3–4 of 6 correct), or good (> 75%; 5–6 of 6 correct). For attitudes, the Likert responses were scored from 1 to 5 and summed, so that higher totals reflected more favourable views. Practice items were scored on an ordinal scale reflecting how adequate the reported behaviour was. A practice score (0–9) was calculated only for participants who provided a substantive response to all three practice items. Participants who selected “not applicable” on one or more of these items were not assigned a practice score. This applied to 13 respondents, yielding a practice-analysis subsample of 60 participants. The self-rated knowledge item and the question on AI awareness were treated descriptively rather than scored, and we set the two against the objective knowledge results in a secondary, perceived-versus-actual comparison [7].

## Data collection

The survey was distributed among participants using Google Form. Participation in the study was voluntary and anonymous, and no identifying information was recorded. The fisrt part of the form explains the study’s purpose, its voluntary nature, and the confidentiality of responses and served as the consent step: only those who agreed could move on to the questionnaire itself.

### Ethical considerations

This study was reviewed and approved by the Research Ethics Committee of King Khalid University, Abha, Saudi Arabia (approval no. KKU-95-2026-14; committee registration HAPO-06-B-001; approved 9 April 2026). The study was conducted in accordance with the Declaration of Helsinki. All participants were informed of the study’s purpose, voluntary nature, and the confidentiality and anonymity of their responses; completion and submission of the anonymous questionnaire were taken as informed consent to participate.

### Statistical analysis

Responses were exported from Google Forms, cleaned, and analyzed in Stata (StataCorp, College Station, TX, USA; version [BE 19.0]). We summarized categorical variables as frequencies and percentages and continuous scores as either mean ± standard deviation or median (interquartile range), depending on their distribution, which we checked with the Shapiro–Wilk test. To explore how knowledge, attitude, and practice categories related to demographic and professional characteristics, we used the chi-square test, or Fisher’s exact test where expected counts were small. Group differences in scores were tested with the Mann–Whitney U or Kruskal–Wallis test when data departed from normality, and with the t-test or one-way ANOVA otherwise. Finally, and in direct answer to the study’s last objective, we examined the interrelationships among the knowledge, attitude, and practice scores using Spearman’s rank correlation. A p-value below 0.05 was treated as significant. The open-ended responses were examined qualitatively through a straightforward thematic reading. All statistical analyses were performed by the author(s) using Stata/BE 19.0 (StataCorp, College Station, TX).

## Results

### Participant characteristics

Seventy-three dental personnel completed the questionnaire. Most were aged 25–34 years (50.7%) or under 25 years (38.4%), and 63.0% were male. The sample spanned the full training-to-practice continuum: 27 (37.0%) were undergraduate dental students (fifth- and sixth-year), 10 (13.7%) were interns, 27 (37.0%) were general dental practitioners, 7 (9.6%) were postgraduate residents, and 2 (2.7%) held an MSc or were dental alumni. General dentistry was the predominant area of practice (60.3%), followed by oral and maxillofacial surgery (13.7%). Respondents were based primarily in Abha (63.0%), with the remainder in Khamis Mushait (15.1%) or working across both cities (21.9%), and were distributed across King Khalid University Hospital (34.2%), private clinics (23.3%), government hospitals including Aseer Central Hospital and AFHSR (20.5%), mixed settings (12.3%), and primary health-care centres (9.6%). Full characteristics are presented in Table 1.

**Table 1:** Demographic and professional characteristics of participants (*n* = 73)

Characteristic	Category	*n* (%)
Age (years)	< 25	28 (38.4)
	25–34	37 (50.7)
	35–44	7 (9.6)
	45–54	1 (1.4)
Gender	Male	46 (63.0)
	Female	27 (37.0)
Professional role	Undergraduate student (5th/6th yr)	27 (37.0)
	Intern	10 (13.7)
	General practitioner	27 (37.0)
	Resident	7 (9.6)
	MSc / alumni	2 (2.7)
Primary specialty	General dentistry	44 (60.3)
	Oral & maxillofacial surgery	10 (13.7)
	Other specialties	19 (26.0)
Work city	Abha	46 (63.0)
	Khamis Mushait	11 (15.1)
	Both	16 (21.9)
Work setting	King Khalid University Hospital	25 (34.2)
	Private dental clinic	17 (23.3)
	Government hospital (ACH/AFHSR)	15 (20.5)
	Mixed	9 (12.3)
	Primary health-care centre	7 (9.6)
FO in undergraduate curriculum	Dedicated subject	12 (16.4)
	Briefly, within another subject	19 (26.0)
	Not included	34 (46.6)
	Do not recall	8 (11.0)
Post-graduation FO training	Formal course/workshop	7 (9.6)
	Self-directed	15 (20.5)
	None	42 (57.5)
	Currently an intern	9 (12.3)

*FO* forensic odontology, *ACH* Aseer Central Hospital, *AFHSR* Armed Forces Hospital Southern Region

One participant whose qualification was “intern” selected a post-graduation-training response other than “currently an intern”; the intern count therefore differs between the qualification row (*n* = 10) and the post-graduation-training row (*n* = 9)

### Prior exposure to forensic odontology

Exposure to forensic odontology during training was limited. Almost half of respondents (46.6%) reported that the subject had not been included in their undergraduate curriculum, and only 16.4% had studied it as a dedicated subject, while 26.0% had encountered it briefly within another course. Post-graduation engagement was similarly low: 57.5% had received no formal or informal training after graduation, and only 9.6% had attended a formal course, workshop, or conference. Consistent with this, self-rated knowledge was modest, with half of participants rating themselves as having poor (30.1%) or no (20.5%) knowledge of the field and only 9.6% rating themselves as excellent.

### Knowledge

Objective knowledge, scored across six single-best-answer items, was low (mean 2.4 ± 1.5 of 6; 40.6 ± 24.5%; median 33.3%). Using Bloom’s classification, 37 respondents (50.7%) demonstrated poor knowledge (< 50%), 29 (39.7%) moderate knowledge (50–75%), and only 7 (9.6%) good knowledge (> 75%). Item-level performance varied widely: respondents were most likely to correctly identify the oral signs of physical abuse (64.4%) and the evidentiary value of dental records (60.3%), and least likely to identify the recommended record-retention period (19.2%), the correct definition of cheiloscopy (23.3%), and the appropriate adult age-estimation method (23.3%) (Table 2).

**Table 2:** Knowledge, attitude, and practice scores, item-level knowledge performance, and inter-domain correlations

Domain	Measure	Value
Knowledge score (0–6)	Mean ± SD	2.4 ± 1.5
	Median (%)	33.3%
Knowledge category	Poor (< 50%)	37 (50.7%)
	Moderate (50–75%)	29 (39.7%)
	Good (> 75%)	7 (9.6%)
Item correct (%)	Evidentiary value of dental records	60.3
	Teeth as a DNA source	53.4
	Oral signs of abuse	64.4
	Adult age estimation	23.3
	Record-retention standard	19.2
	Cheiloscopy	23.3
Attitude score (4–20)	Mean ± SD (Cronbach’s α = 0.93)	14.8 ± 4.7
Practice score (0–9; *n* = 60)	Mean ± SD	5.7 ± 2.1
Correlations (Spearman, *n* = 60)	Knowledge–Attitude	ρ = 0.45, *p* = 0.0004
	Knowledge–Practice	ρ = 0.26, *p* = 0.042
	Attitude–Practice	ρ = 0.13, *p* = 0.33

*SD* standard deviation

Correlations are Spearman's rho; practice-related analyses are based on *n* = 60

**Fig. 1:**
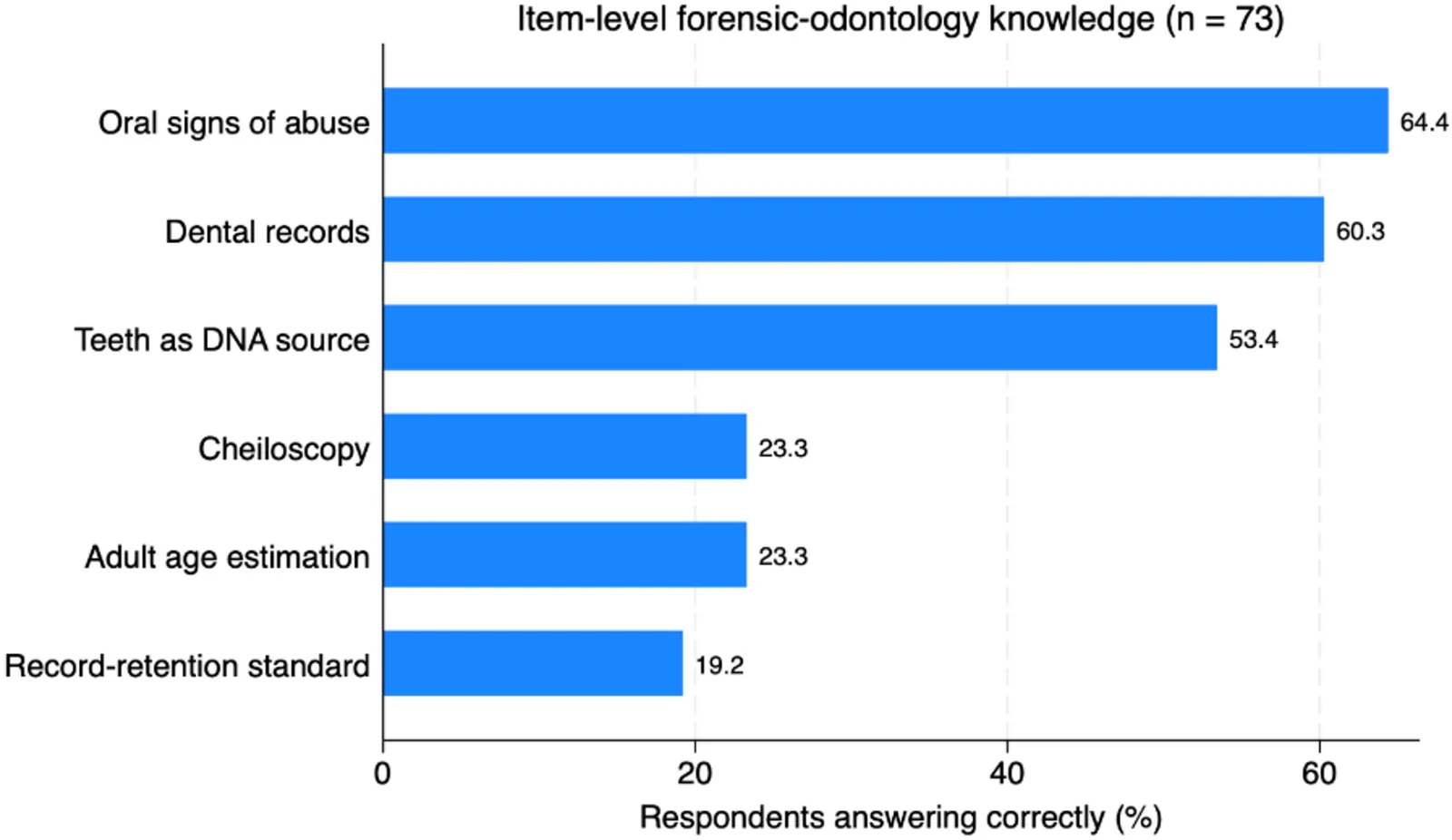
Proportion of respondents (*n* = 73) correctly answering each forensic-odontology knowledge item

When asked to identify recognized applications of forensic odontology, most respondents correctly selected human identification (80.8%), dental age estimation (69.9%), and bite-mark analysis (60.3%), whereas the distractor option (routine periodontal treatment) was selected by only 8.2%. Awareness of artificial intelligence applications in forensic dentistry was limited: 32.9% reported familiarity, 28.8% had heard of it without detail, and 38.4% were either unaware or skeptical of its role. Item-level knowledge performance is shown in Fig. 1.

### Attitudes

Attitudes, measured on four pro-field five-point Likert items, were consistently favourable (mean 14.8 ± 4.7 of 20; median 16), and the attitude scale showed high internal consistency (Cronbach’s α = 0.93). Respondents broadly agreed that forensic odontology is an important and undervalued field, that practitioners bear a professional responsibility to maintain forensic-quality records, that the subject should be a dedicated component of the undergraduate curriculum, and that they would participate in continuing professional development on the topic. Favourable attitudes were uniform across the sample, with no significant differences by gender, professional role, work setting, clinical experience, or city (all *p* > 0.05).

### Record-keeping practices

Among the 60 participants who provided substantive responses to all three practice items (13 respondents selected “not applicable” on at least one item and were therefore excluded from the practice-score analysis), reported practices were moderate (mean practice score 5.7 ± 2.1 of 9; median 6). Comprehensive documentation of dental history was reported by 38.4% of respondents “always” and 32.9% “usually,” while approximately one-quarter did so rarely or not at all. Baseline radiographs were routinely obtained for new patients by 56.2%, and 23.3% obtained them only when clinically indicated. Record retention was the weakest domain: only 19.2% reported retaining records indefinitely or for at least ten years, whereas 34.2% did not know their facility’s retention period and a further 30.1% retained records for less than ten years.

The clinical relevance of these gaps was evident in respondents’ direct experience. Overall, 43.8% (32/73) reported having encountered a situation in which dental records were requested for forensic, medico-legal, or insurance purposes; in 17.8% of the full sample the requested records were incomplete or unavailable, whereas 26.0% had been able to provide complete records. A further 20.5% were unsure, and 35.6% had never encountered such a request. The most frequently cited barriers to adequate record-keeping were a lack of awareness of the medico-legal value of records (60.3%) and the absence of a standardised or digital record-keeping system (43.8%), followed by time constraints (38.4%) and the absence of an institutional mandate (28.8%).

### Associations and inter-domain correlations

Although categorical knowledge level differed by sex (χ² *p* = 0.001), this result rested on only seven high-scoring respondents and was not corroborated by the continuous knowledge score (Mann–Whitney *p* = 0.098); it should therefore be regarded as a preliminary signal requiring confirmation in larger samples rather than a robust sex difference. Knowledge scores also differed significantly by professional role (Kruskal–Wallis *p* = 0.033) and clinical experience (*p* = 0.010), with students and interns tending to outperform general practitioners. Practice scores differed significantly by work setting (*p* = 0.039) but not by other factors, while attitudes did not differ across any subgroup.

Addressing the study’s principal objective, Spearman correlations among the three domains (*n* = 60) showed a moderate, statistically significant positive correlation between knowledge and attitude (ρ = 0.45, *p* = 0.0004) and a weak but significant positive correlation between knowledge and practice (ρ = 0.26, *p* = 0.042). The correlation between attitude and practice was weak and non-significant (ρ = 0.13, *p* = 0.33). Thus, respondents with greater knowledge tended to hold more favourable attitudes and to report somewhat better record-keeping, whereas favourable attitudes alone were not associated with practice (Table 2).

## Discussion

This multi-centre survey of 73 dental students, interns, and practitioners across the Aseer region examined knowledge, attitudes, and practices relating to forensic odontology and dental record-keeping. The central finding is a clear dissociation between attitudes and knowledge: respondents held strongly favourable attitudes toward the field (mean 14.8 of 20; Cronbach’s α = 0.93) yet demonstrated poor objective knowledge (mean 40.6%; only 9.6% reaching the “good” threshold and more than half classified as poor). The deficit in this population is therefore not one of motivation or perceived relevance but of knowledge and training. This dissociation between favourable perception and limited objective knowledge mirrors a national Saudi survey in which dentists’ perceived competence exceeded their actual knowledge of forensic odontology [7]. This pattern is consistent with earlier Saudi work; Salam et al. reported high awareness of the importance of forensic dentistry alongside limited practical preparedness [4], and a Riyadh-based study found that only 40.9% of undergraduates, versus 75% of postgraduates, recognised teeth as a source of DNA [6], echoing the knowledge gradient observed here. Comparable shortfalls have been reported among dental professionals in western Saudi Arabia [8] and in the wider population, where public awareness of forensic odontology and of a national dental registry remained limited [9].

The knowledge deficit appears rooted in education rather than disposition. Almost half of respondents (46.6%) reported that forensic odontology was absent from their undergraduate curriculum, only 16.4% had studied it as a dedicated subject, and 57.5% had received no post-graduation training. The weakest individual items reinforce this interpretation: only 19.2% correctly identified the recommended record-retention period and 34.2% did not know their own facility’s retention practice, while fewer than one-quarter correctly defined cheiloscopy or the adult age-estimation method. These are precisely the topics that formal curricular exposure, rather than general clinical experience, would be expected to address; cheiloscopy denotes the study of lip prints for identification [10], while the pulp-to-tooth area ratio described by Cameriere et al. is a recognised method for adult dental age estimation [11]. Weak record-keeping has likewise been documented internationally: a study of general dental practitioners in Pune, India, found that 30% kept no records and most retained them for two years or less [12], and a large Indian survey reported that, although 97% kept records, only 13.4% were complete and most were unaware of recommended retention periods [13]. The observation that students and interns outperformed general practitioners (Kruskal–Wallis *p* = 0.033 by role; *p* = 0.010 by experience) is best explained by the recency of their theoretical education, and suggests that knowledge, once acquired, is not durably reinforced in routine practice without structured continuing education.

The correlation structure among the three domains carries direct practical implications. Knowledge correlated moderately with attitudes (ρ = 0.45, *p* = 0.0004) and weakly with practice (ρ = 0.26, *p* = 0.042), whereas attitudes and practice were unrelated (ρ = 0.13, *p* = 0.33). Favourable attitudes did not, by themselves, translate into better record-keeping; knowledge was the only domain associated with practice. This argues that interventions aimed solely at raising awareness or enthusiasm are unlikely to change behaviour, and that targeted knowledge- and skills-based training is the more promising lever. The barriers most frequently cited by respondents — a lack of awareness of the medico-legal value of records (60.3%) and the absence of a standardised or digital record-keeping system (43.8%) — point to a dual remedy combining educational and systems-level reform. A comparable awareness gap was reported among Lebanese dentists, where only a minority were familiar with the legal requirements for maintaining dental records [14].

The deficits identified here also reflect system-level factors. In the absence of a standardized, enforceable framework and a shared digital infrastructure for dental record creation and retention, individual awareness is unlikely to translate into consistent practice. Establishing clear institutional retention policies, interoperable electronic dental records, and a national dental registry coupled with professional-body guidance would provide the structural support required to convert favourable attitudes into reliable, forensically usable documentation.

These gaps carry particular weight in the Saudi context, and the present data indicate that the need is not hypothetical. Nearly half of respondents (43.8%) had already encountered a request for dental records for forensic, medico-legal, or insurance purposes, and in 17.8% of the full sample the records were incomplete or unavailable when needed — a locally observed manifestation of the documentation gap. The Kingdom further hosts recurrent mass gatherings during Hajj and Umrah, and the Aseer region records substantial road-traffic mortality, both generating scenarios in which dental identification and disaster-victim identification (DVI) may be required [15, 16]. When good-quality antemortem records are available, forensic odontology classically contributes to the identification of a substantial proportion of victims in mass-fatality incidents [16]. A workforce that is favourably disposed toward forensic odontology but unable to maintain forensic-quality records, and largely unaware of retention standards, represents a preventable vulnerability in regional medico-legal preparedness; comparable educational shortfalls have recently been described among dental students and practitioners in Egypt [17]. The low awareness of artificial-intelligence applications (only 32.9% familiar) indicates a further opportunity to introduce contemporary identification tools alongside foundational training, as AI-based methods are increasingly applied to dental age estimation, bite-mark analysis, and record comparison [18]. A recent systematic review reported promising but still heterogeneous diagnostic performance for these tools [18], suggesting that curricular and continuing-education initiatives should incorporate emerging AI methods alongside foundational forensic principles rather than treat them as a substitute for them.

The finding that all seven respondents in the “good” knowledge category were female (χ² *p* = 0.001), despite a male-majority sample, should be interpreted cautiously. The continuous knowledge score did not differ significantly by gender (Mann–Whitney *p* = 0.098), so the categorical association rests on a small number of high-scoring respondents and may reflect chance or unmeasured confounding rather than a genuine gender effect; it warrants confirmation in larger samples before any inference is drawn.

### Strengths and limitations

To our knowledge, this is the first study to assess forensic-odontology knowledge, attitudes, and practices among dental personnel in the Aseer region, and it draws on multiple institutions including a university hospital, government hospitals, and private clinics. The knowledge instrument used single-best-answer items with objectively defined correct responses, and the attitude scale demonstrated high internal consistency. Several limitations should be acknowledged. The sample was a regional convenience sample of modest size (*n* = 73), limiting subgroup power and generalisability beyond Aseer, and some demographic and professional strata contained few respondents. The inclusion of undergraduate students and interns alongside qualified practitioners produced a heterogeneous population, and the practice analysis was necessarily restricted to the 60 respondents who provided a substantive response to all three practice items, as the remaining 13 selected “not applicable” on at least one item. Practices were self-reported and therefore subject to recall and social-desirability bias, and the cross-sectional design precludes causal inference. No a priori sample-size calculation was performed, and the convenience-sampling strategy carries a risk of selection bias; the sample may therefore not be representative even of the Aseer dental workforce. Because the questionnaire was distributed through shared professional channels without a closed denominator, a response rate could not be calculated. Finally, the single-region sampling frame means the findings should be regarded as hypothesis-generating for the wider Saudi context rather than nationally representative.

## Conclusions

In this preliminary, single-region study based on a convenience sample of 73 dental personnel, participants held favourable attitudes toward forensic odontology but demonstrated limited knowledge and inconsistent record-keeping practices, with knowledge rather than attitude being the factor most closely associated with practice. The findings identify a specific and addressable educational gap: forensic odontology and forensic-quality documentation are insufficiently embedded in undergraduate curricula and post-graduate training. Integrating structured forensic-odontology teaching into the dental curriculum, providing continuing professional development for practising dentists, and implementing standardised or digital record-keeping systems with clear retention policies would address the deficits identified here and strengthen regional medico-legal and disaster-victim-identification preparedness. These findings should be regarded as exploratory and hypothesis-generating; the recommendations that follow warrant confirmation in larger, multi-region studies before broad implementation.

## Supplementary Information

Supplementary Material 1.

## Data Availability

The datasets generated and analyzed during the current study are available from the corresponding author on reasonable request.
