# Supplementary material for: Behind the smile: forensic odontology knowledge, attitudes, and practices among dental students, interns, and practitioners in the Asir region, Saudi Arabia: a cross-sectional study
Source: BMC Oral Health. 2026 Jul 31;26:2110. doi: 10.1186/s12903-026-09465-2 (PMC13712062; doi:10.1186/s12903-026-09465-2)
Supplement: Supplementary file 1 [file 12903_2026_9465_MOESM1_ESM.pdf]

Behind the Smile: Forensic Odontology Knowledge, Attitudes, and Practices Among Dental Practitioners, Residents, and Interns in Asir region, Saudi Arabia

73 responses

Publish analytics

Demographic and Professional Background

Your age group?

Copy chart

73 responses

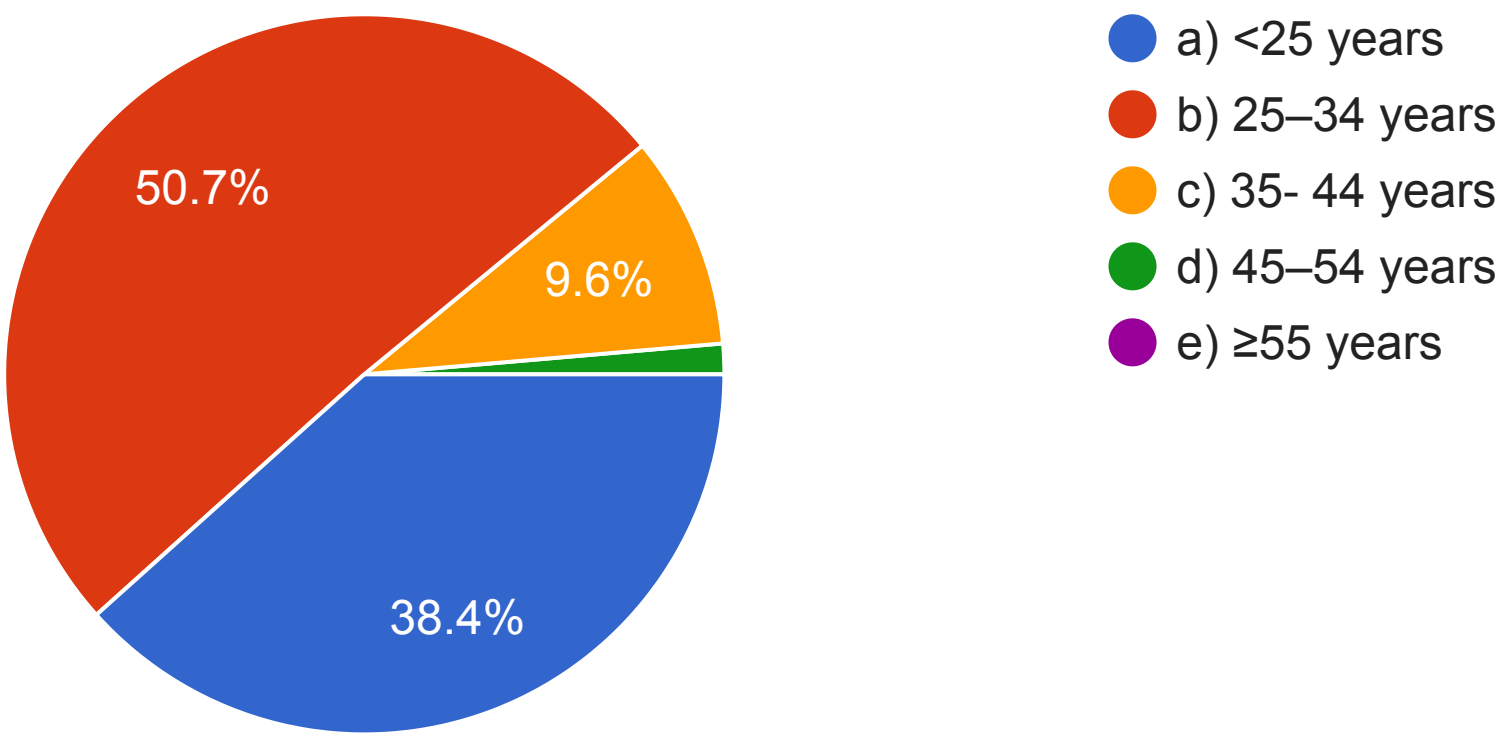

Your gender?

Copy chart

73 responses

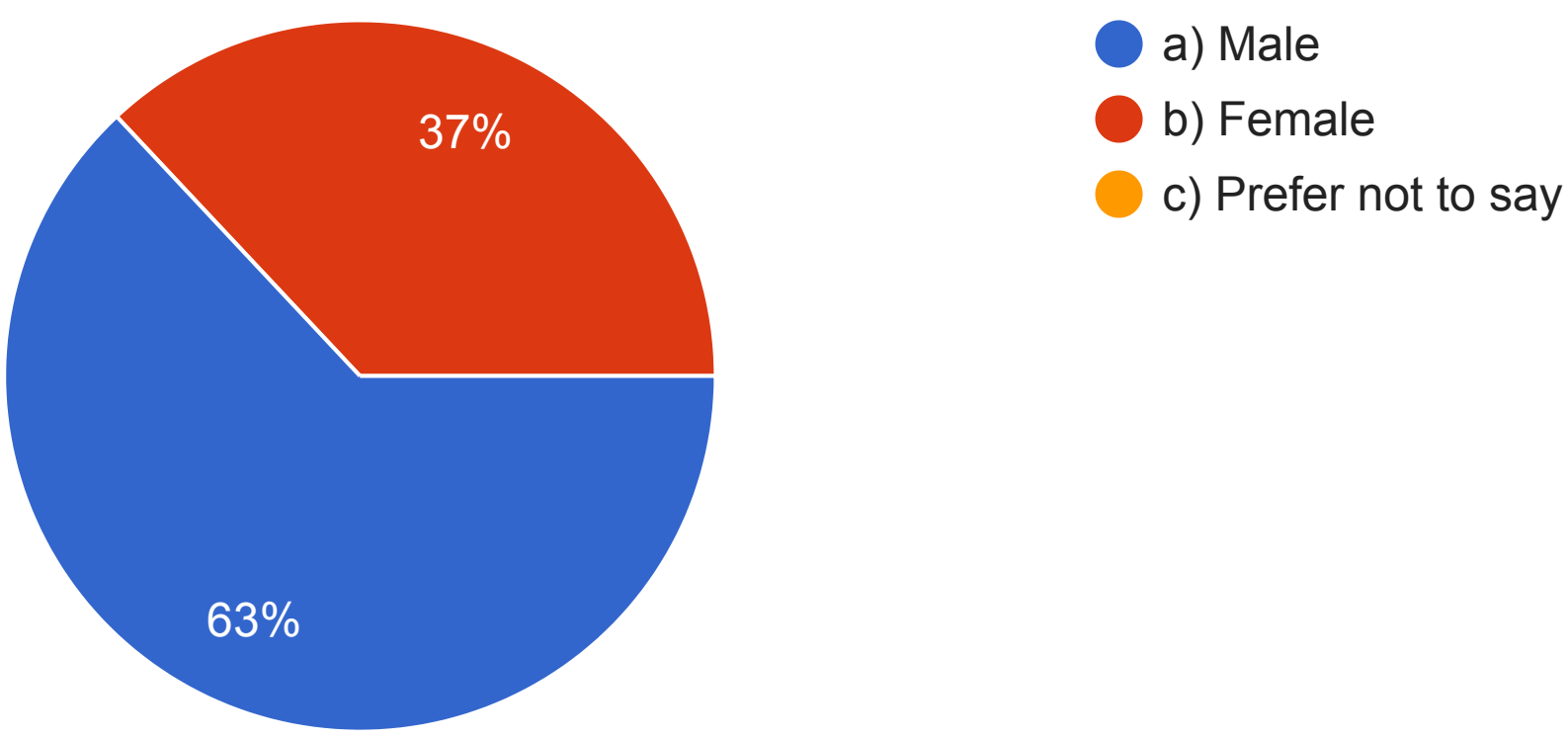

Your highest dental qualification?

Copy chart

73 responses

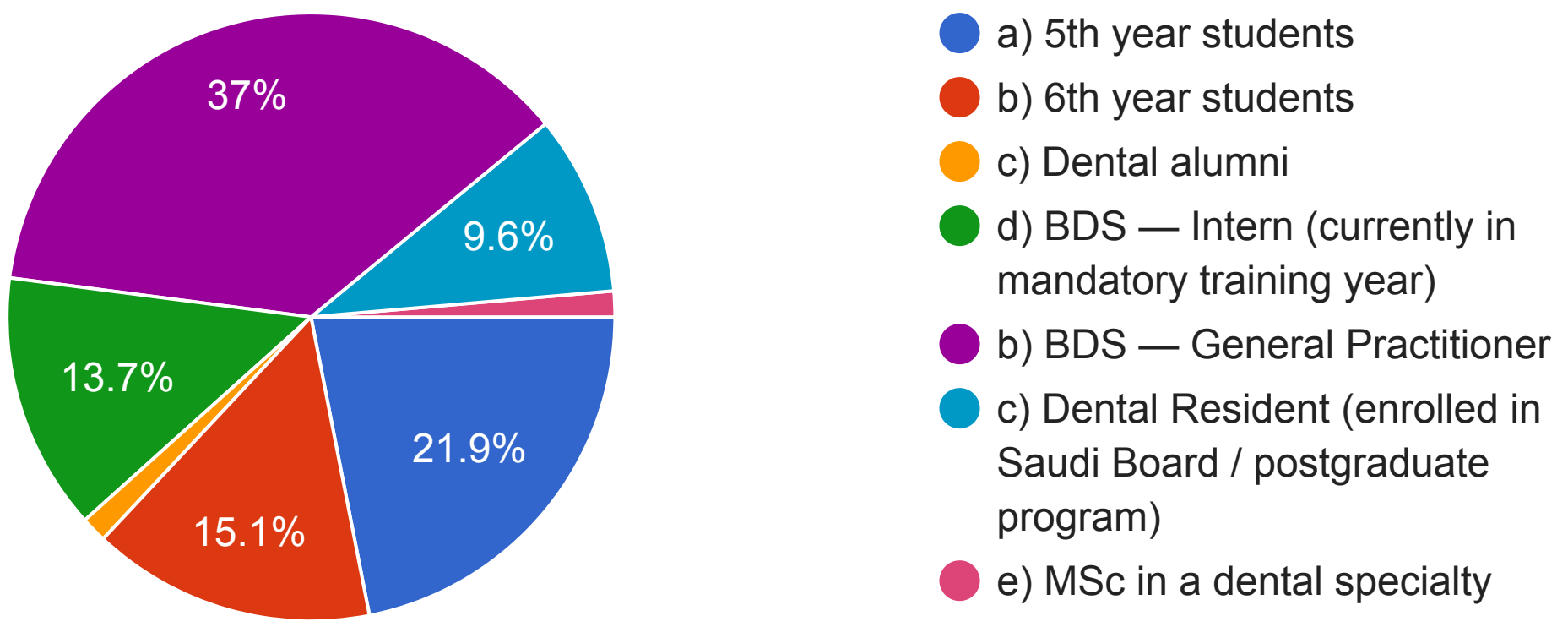

What is your primary dental specialty or area of practice?

Copy chart

73 responses

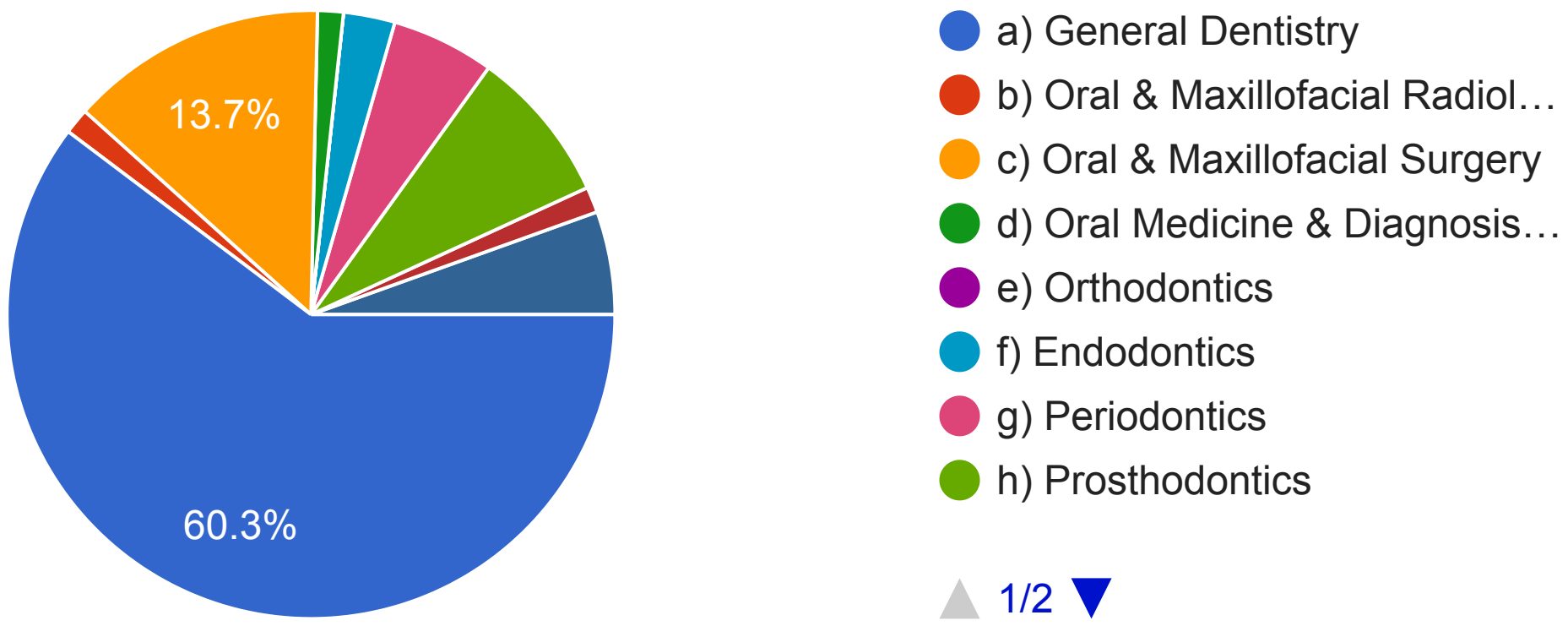

Years of clinical experience you have (post-BDS, excluding intern year)?

Copy chart

73 responses

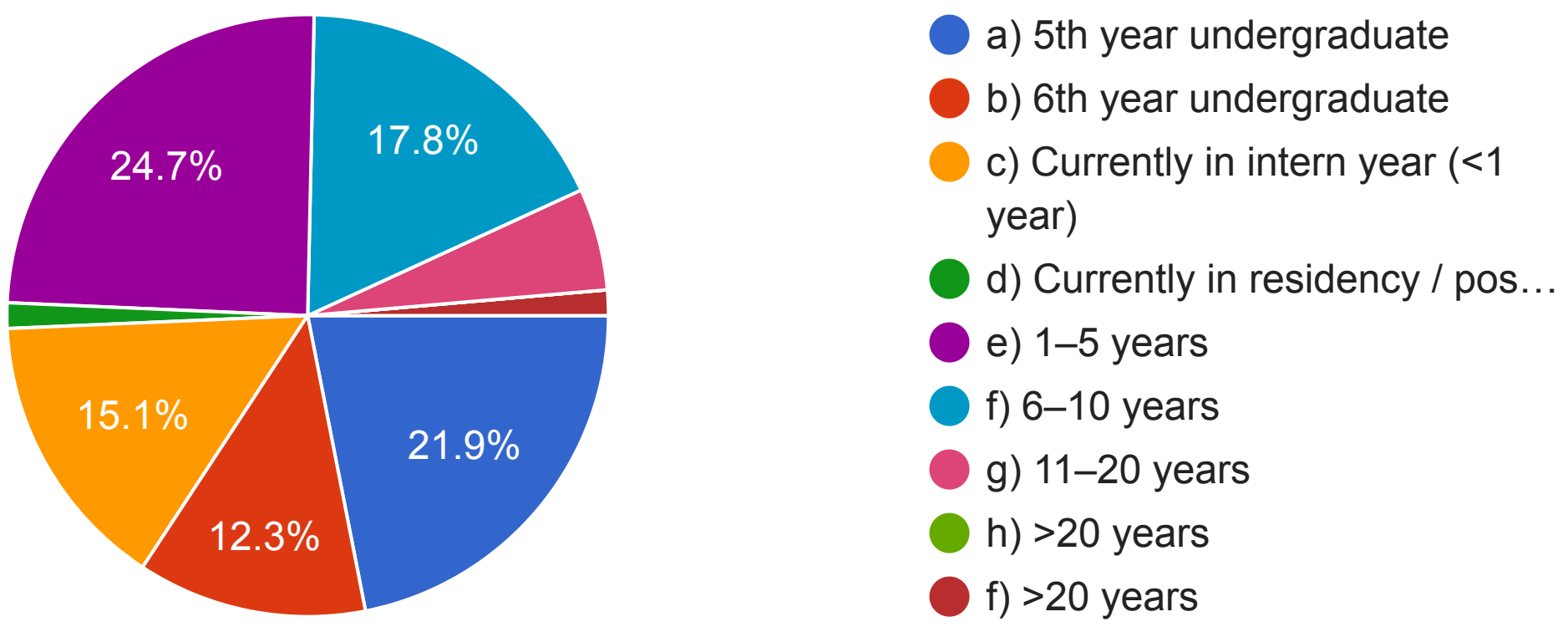

Your primary work city?

Copy chart

73 responses

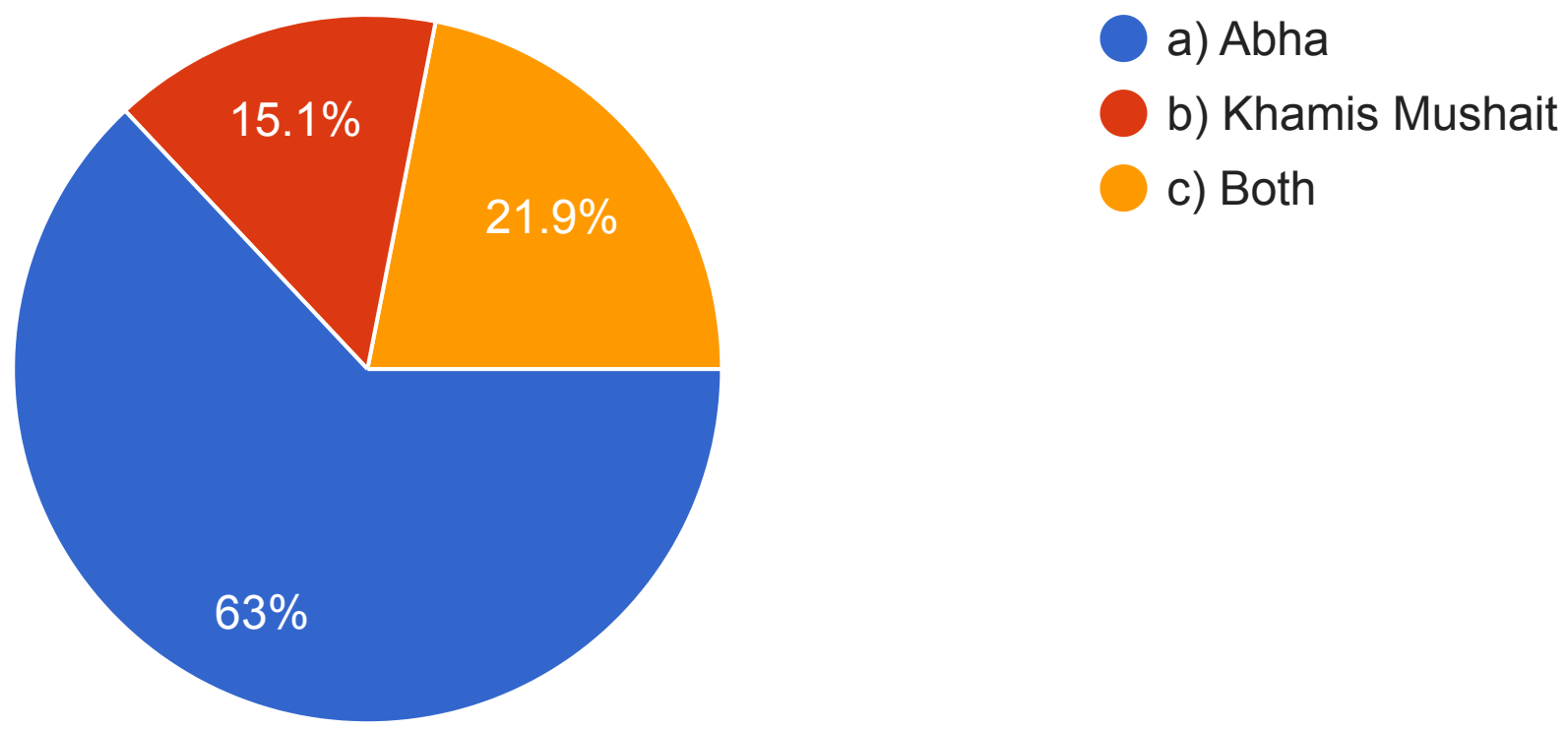

Your primary work setting?

Copy chart

73 responses

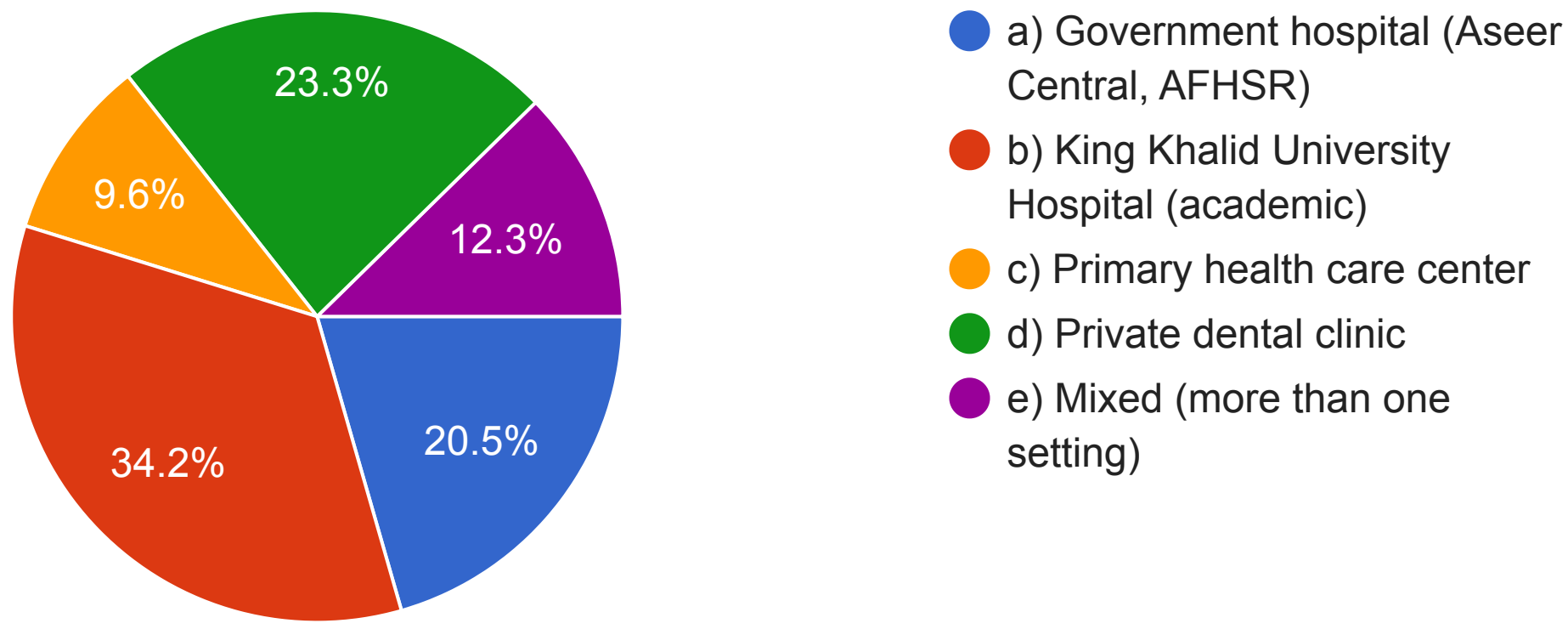

Prior Exposure to Forensic Odontology n

Was forensic odontology taught as part of your undergraduate dental curriculum?

Copy chart

73 responses

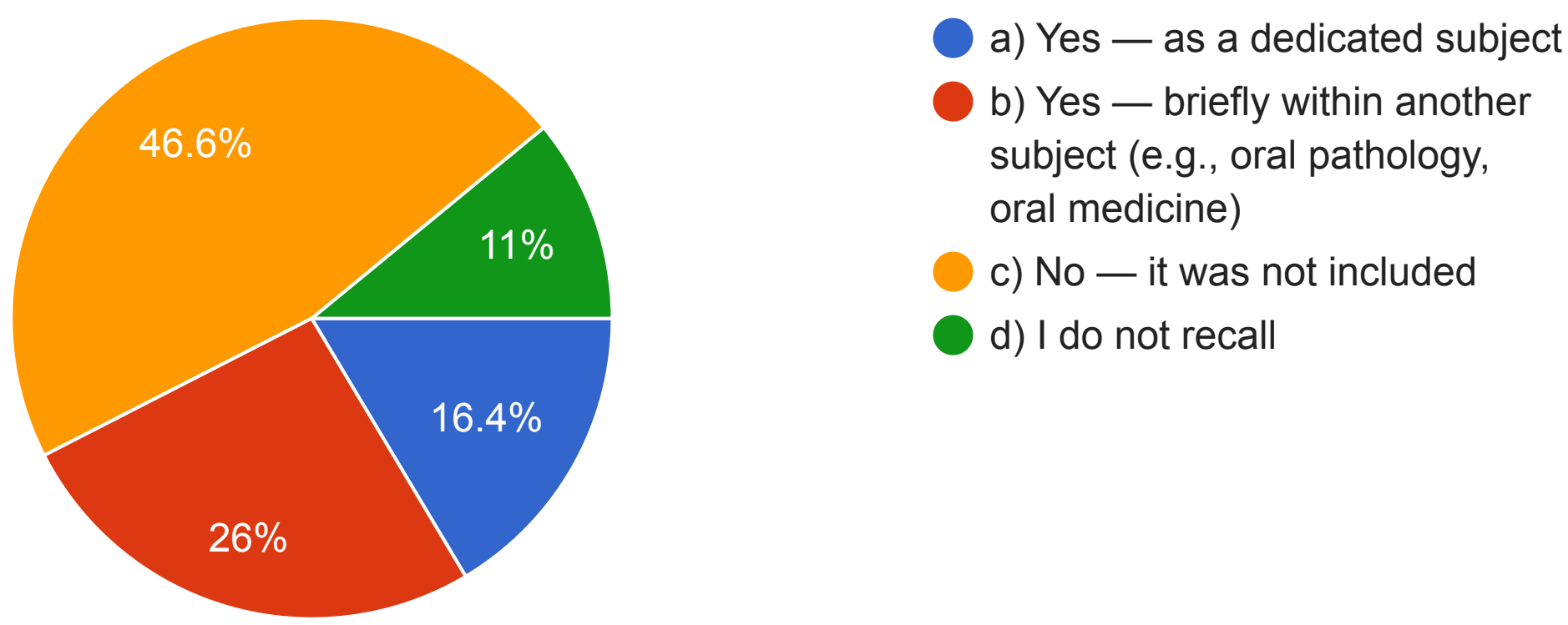

Copy chart

Have you received any formal training in forensic odontology after graduation?

73 responses

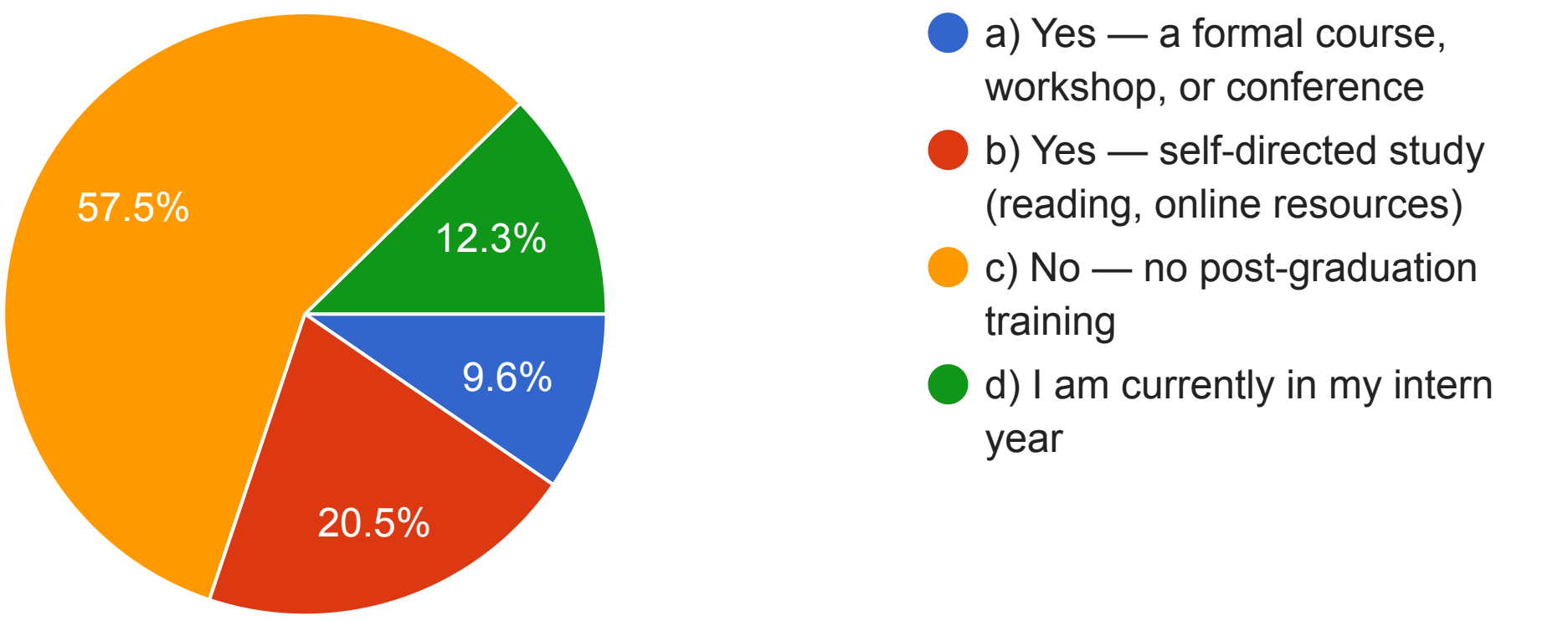

Copy chart

How would you rate your current knowledge of forensic odontology?

73 responses

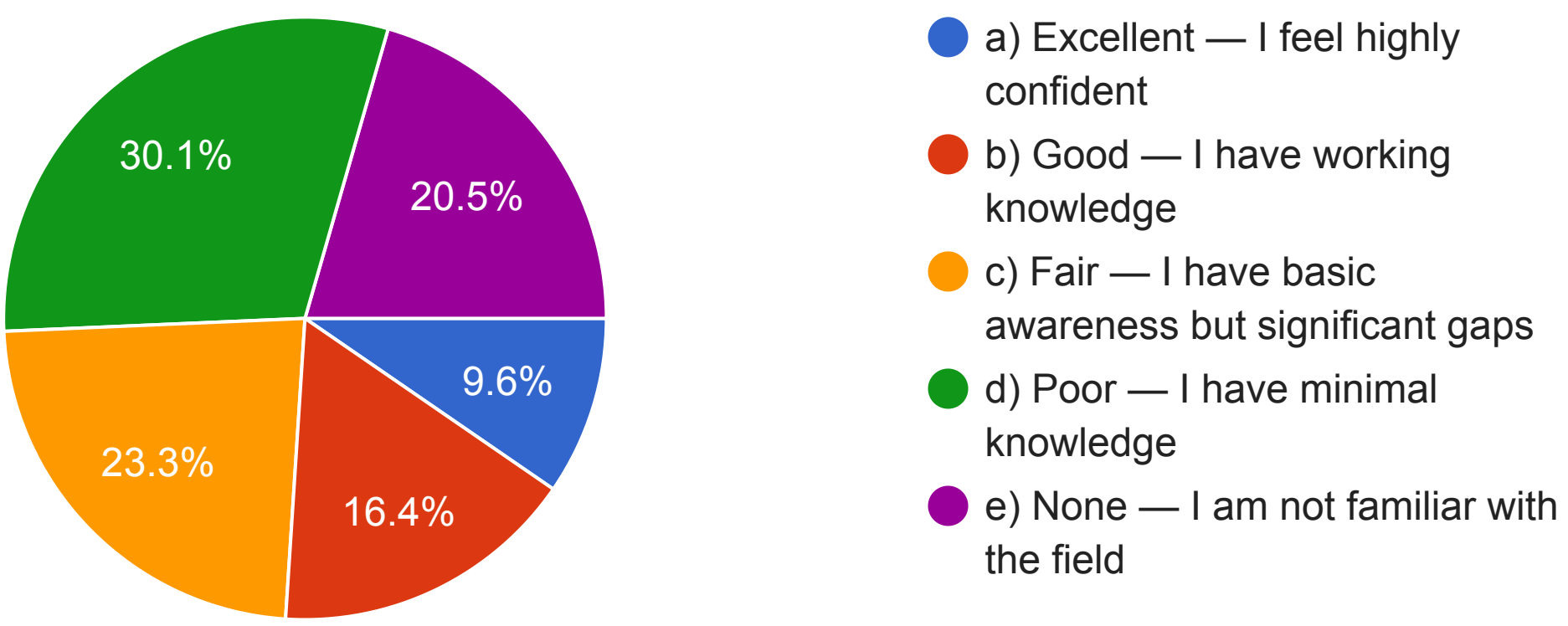

Knowledge of Forensic Odontology

Which of the following are recognized applications of forensic odontology? (Select ALL that apply)

Copy chart

73 responses

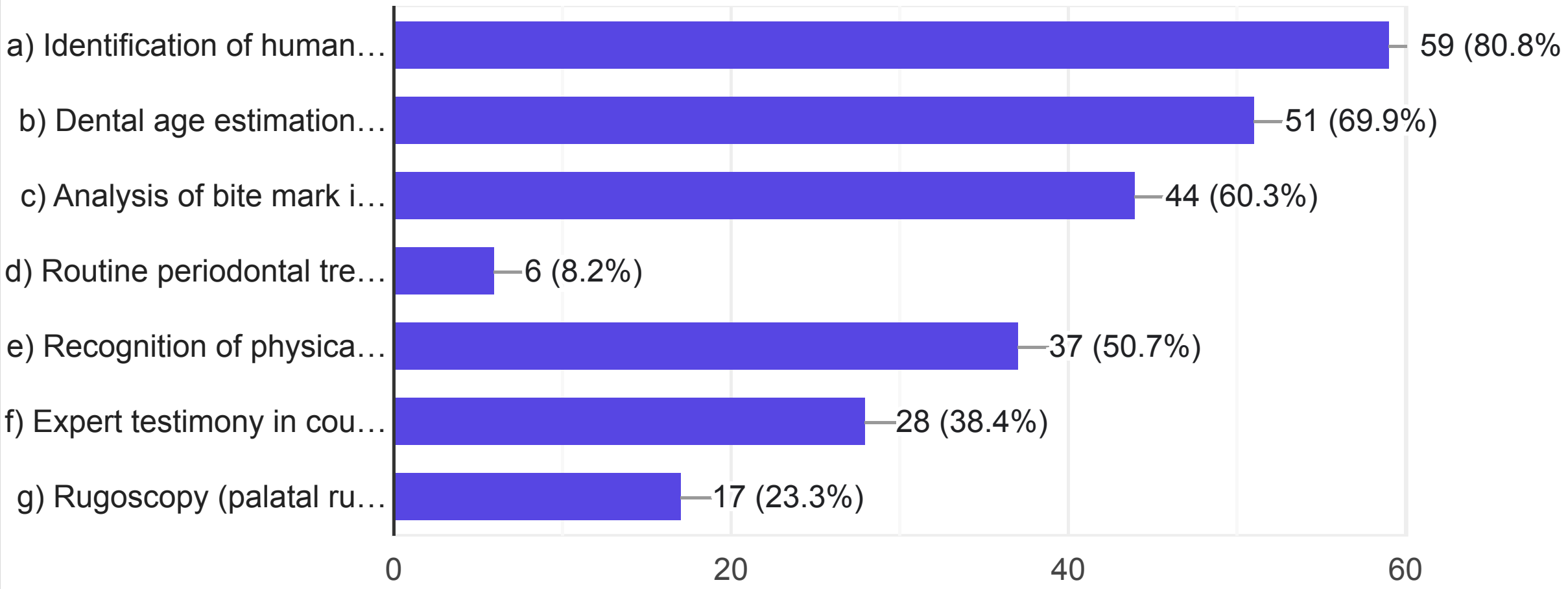

In forensic victim identification, dental records are considered:

Copy chart

73 responses

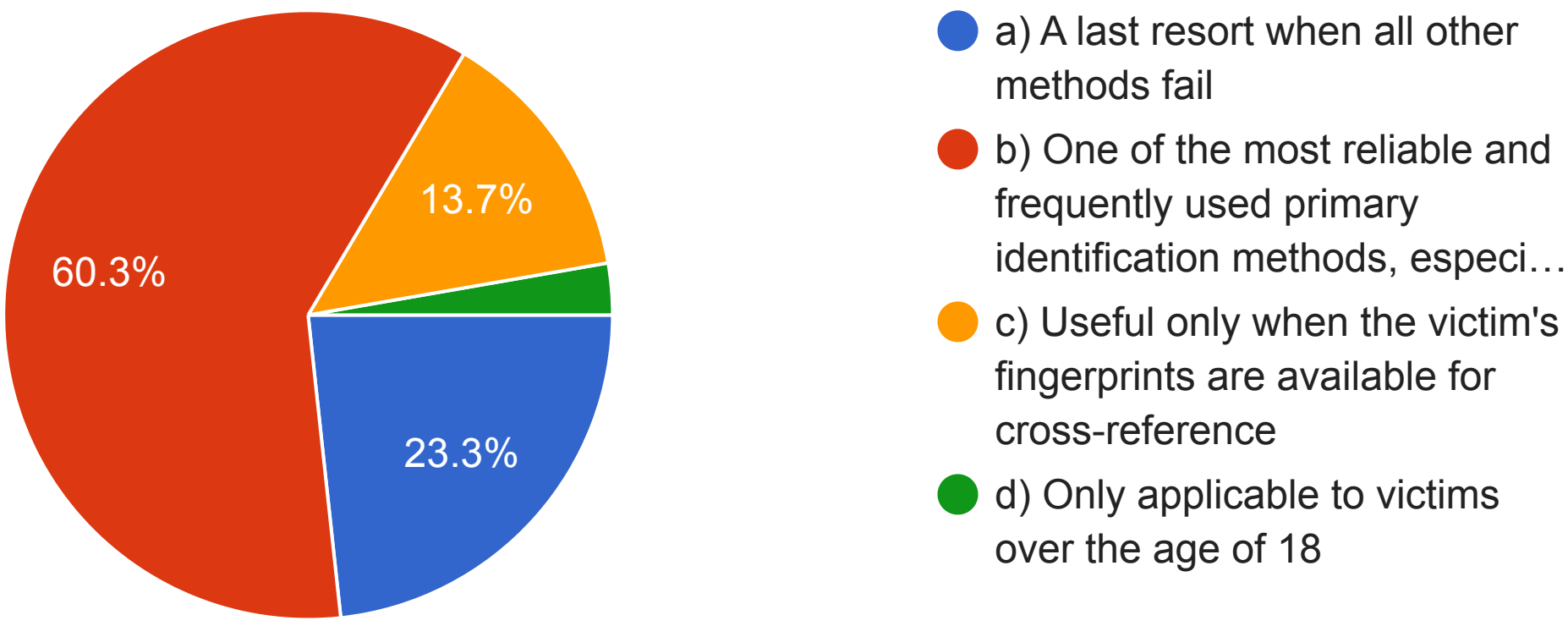

Teeth can serve as a source of DNA for forensic identification.

Copy chart

73 responses

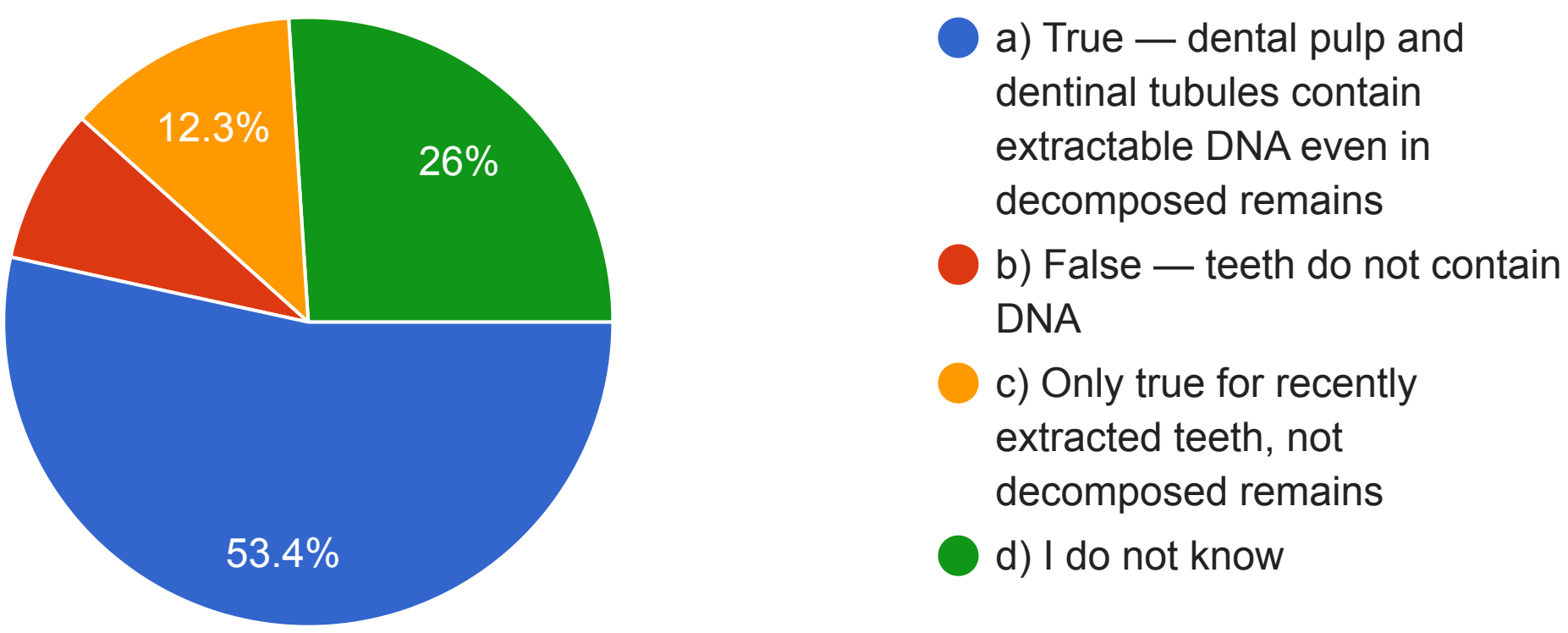

Which method is most commonly used for forensic dental age estimation in adults when no birth records are available?

Copy chart

73 responses

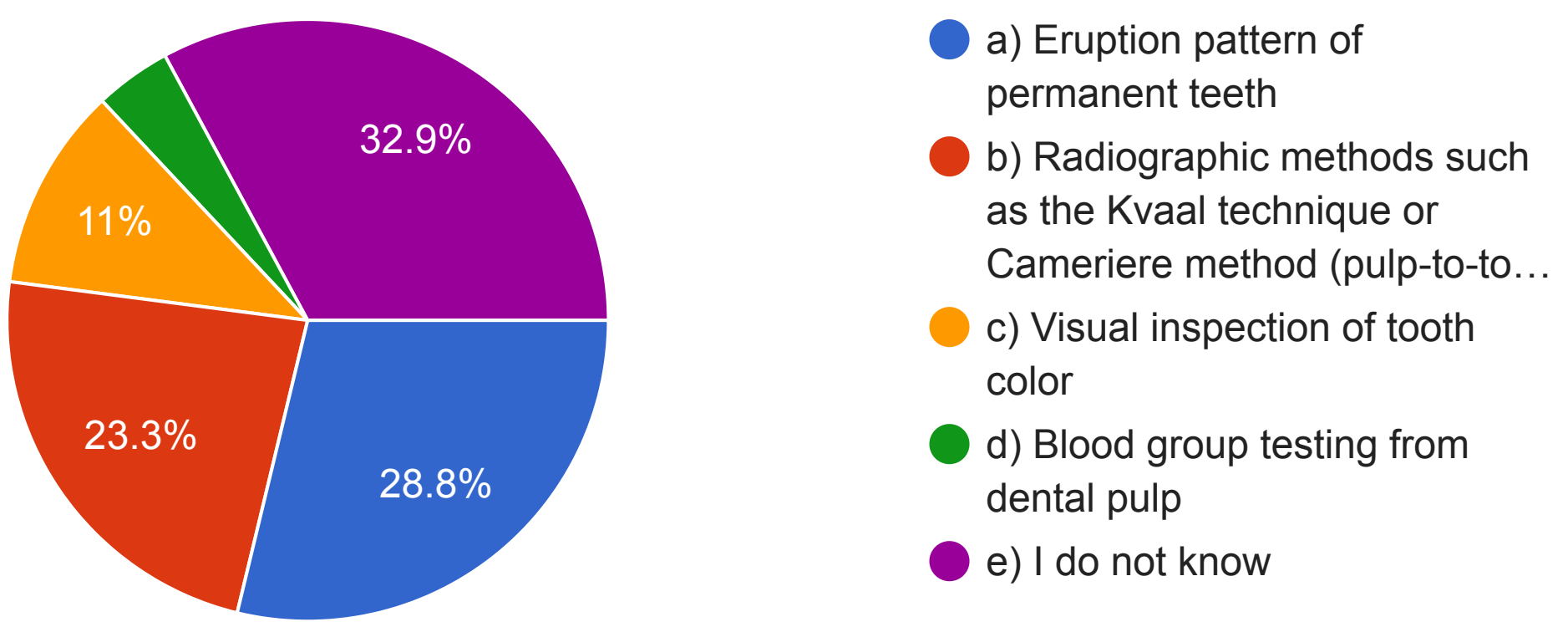

Forensic dentistry plays a role in cases of child abuse. Which of the following oral/perioral signs may indicate physical abuse?

Copy chart

73 responses

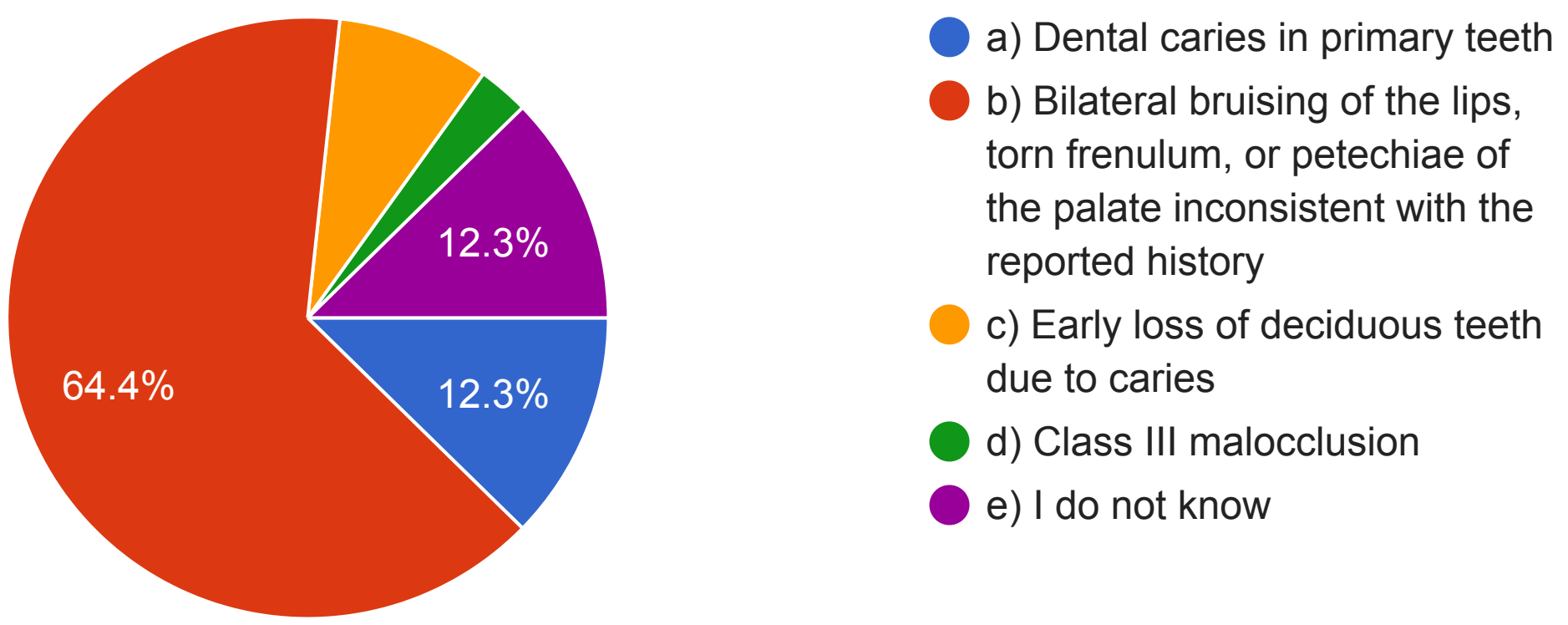

How long should dental records (radiographs, clinical notes, study models) be retained according to international best practice and medico-legal standards applicable to dentistry?

Copy chart

73 responses

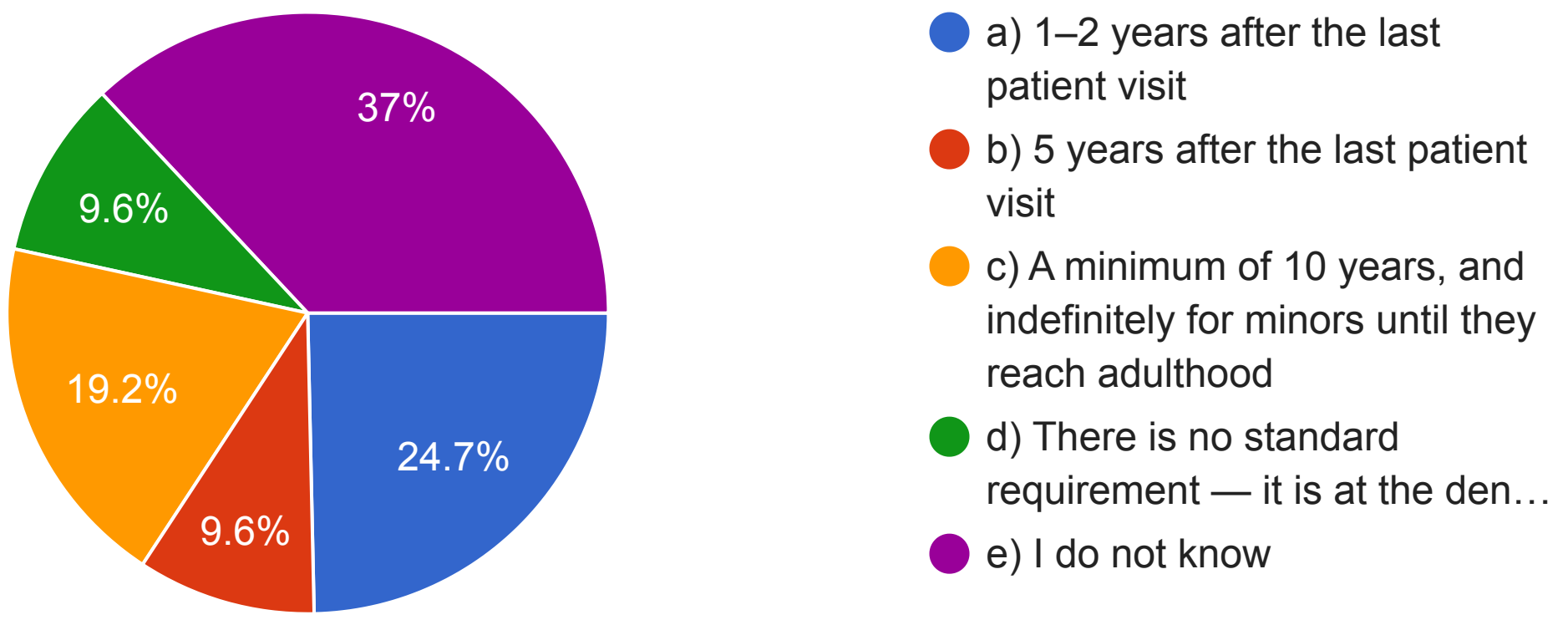

Copy chart

Cheiloscopy refers to:

73 responses

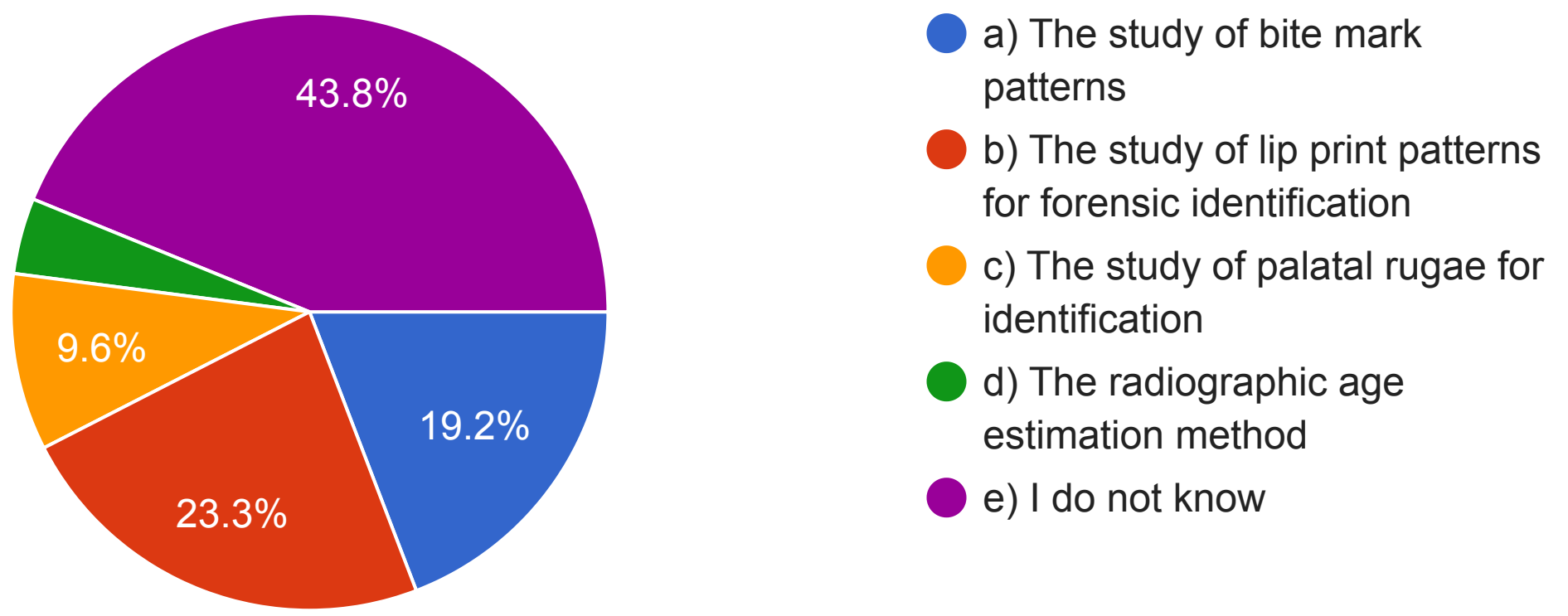

Copy chart

Are you aware that artificial intelligence (AI) tools are now being developed and validated for forensic dental age estimation and identification?

73 responses

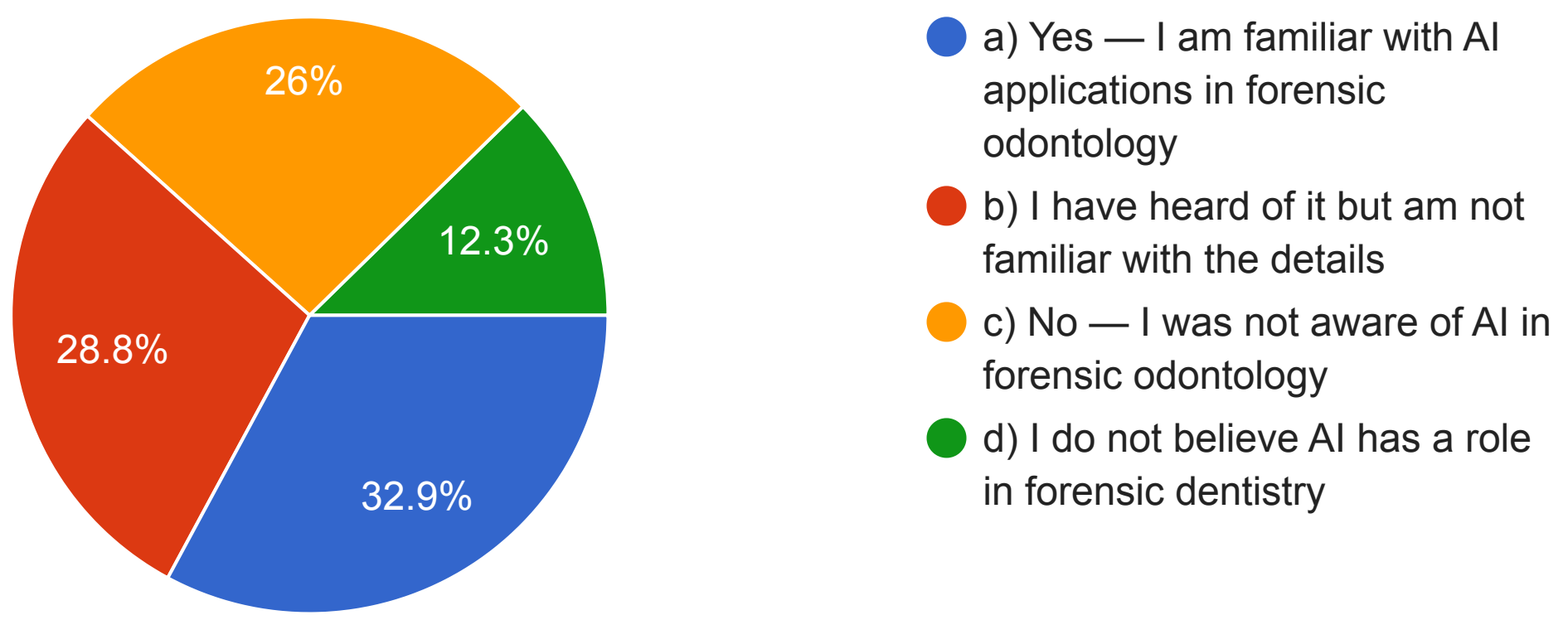

Attitudes Toward Forensic Odontology

Copy chart

Forensic odontology is an important and undervalued field of dentistry in Saudi Arabia.

73 responses

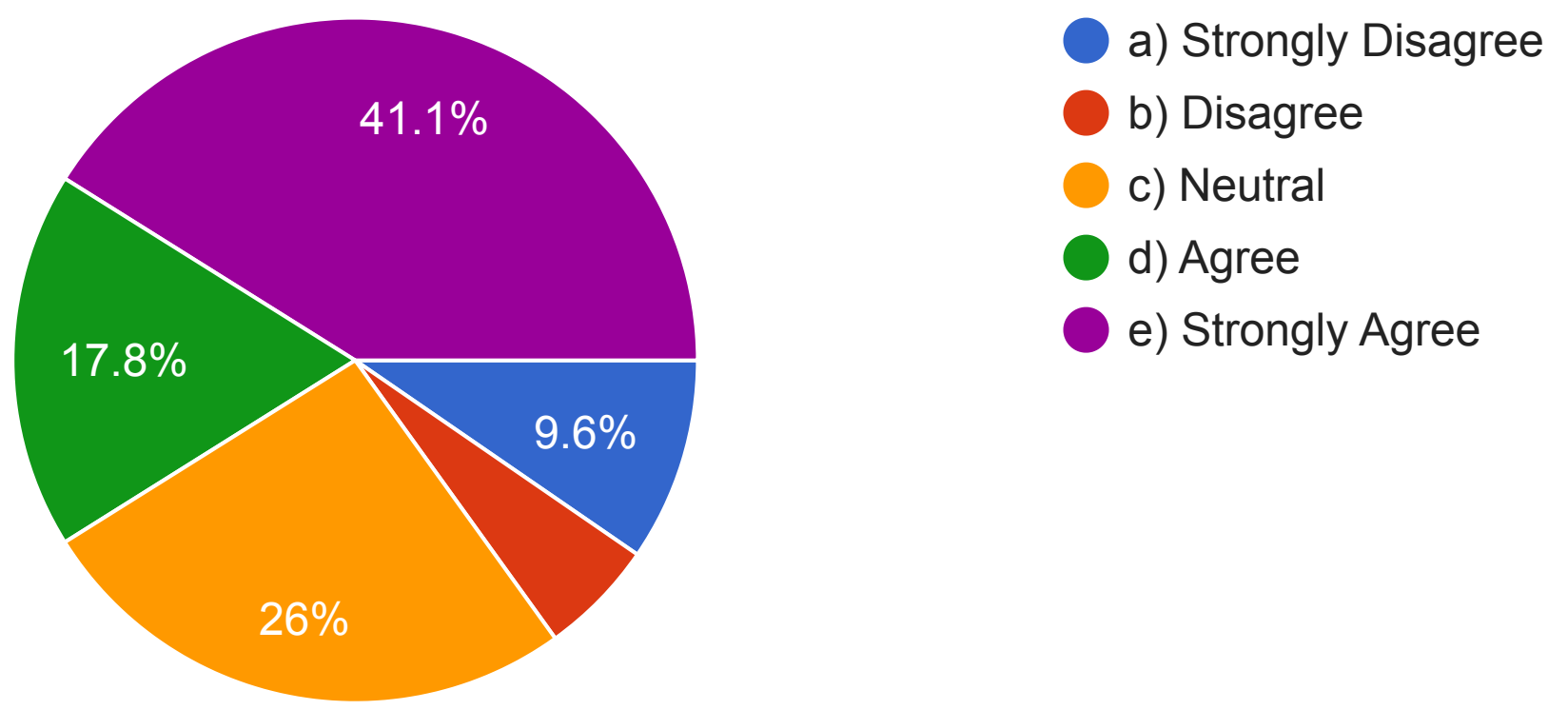

Dental practitioners have a professional responsibility to maintain complete and accurate dental records that could serve forensic purposes.

Copy chart

73 responses

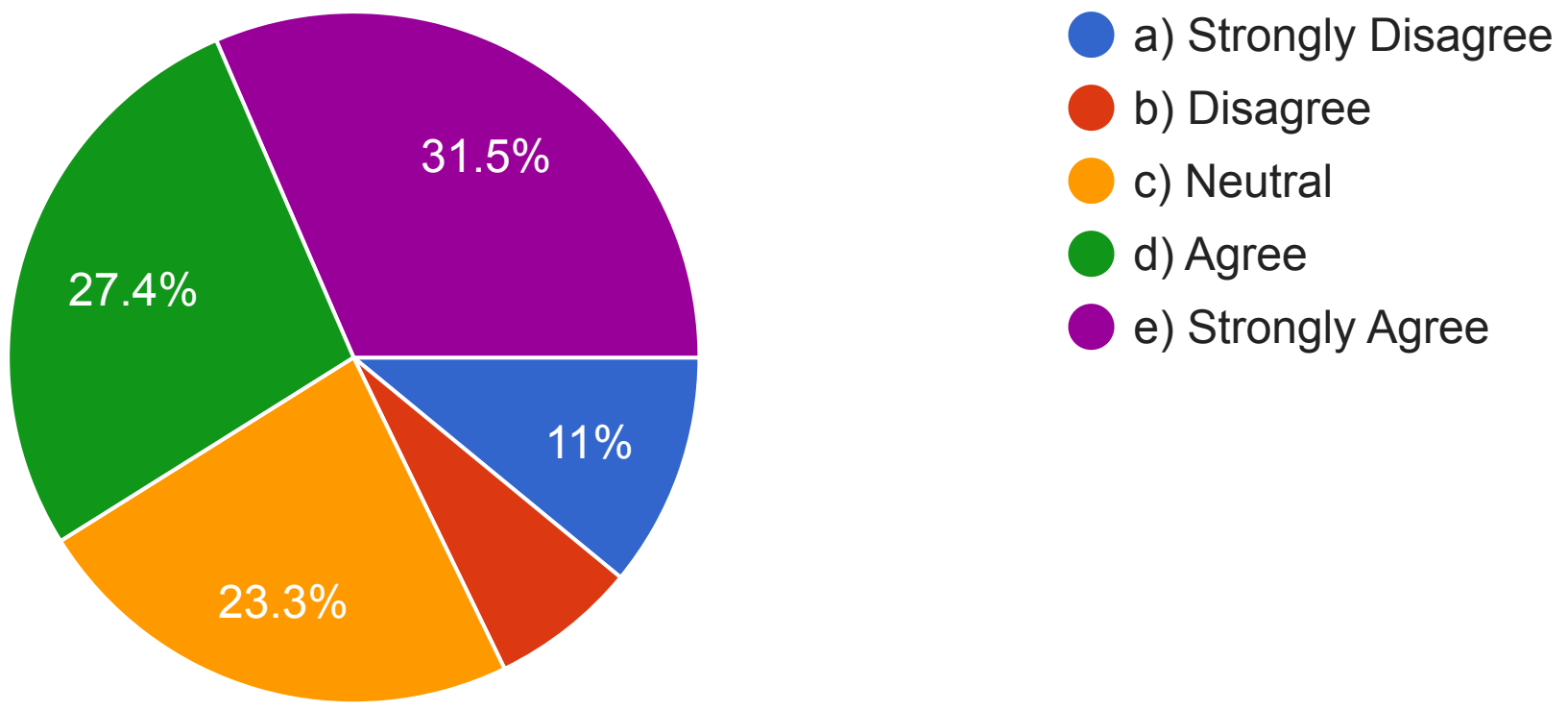

Forensic odontology should be included as a dedicated subject in the Saudi dental undergraduate curriculum (BDS program).

Copy chart

73 responses

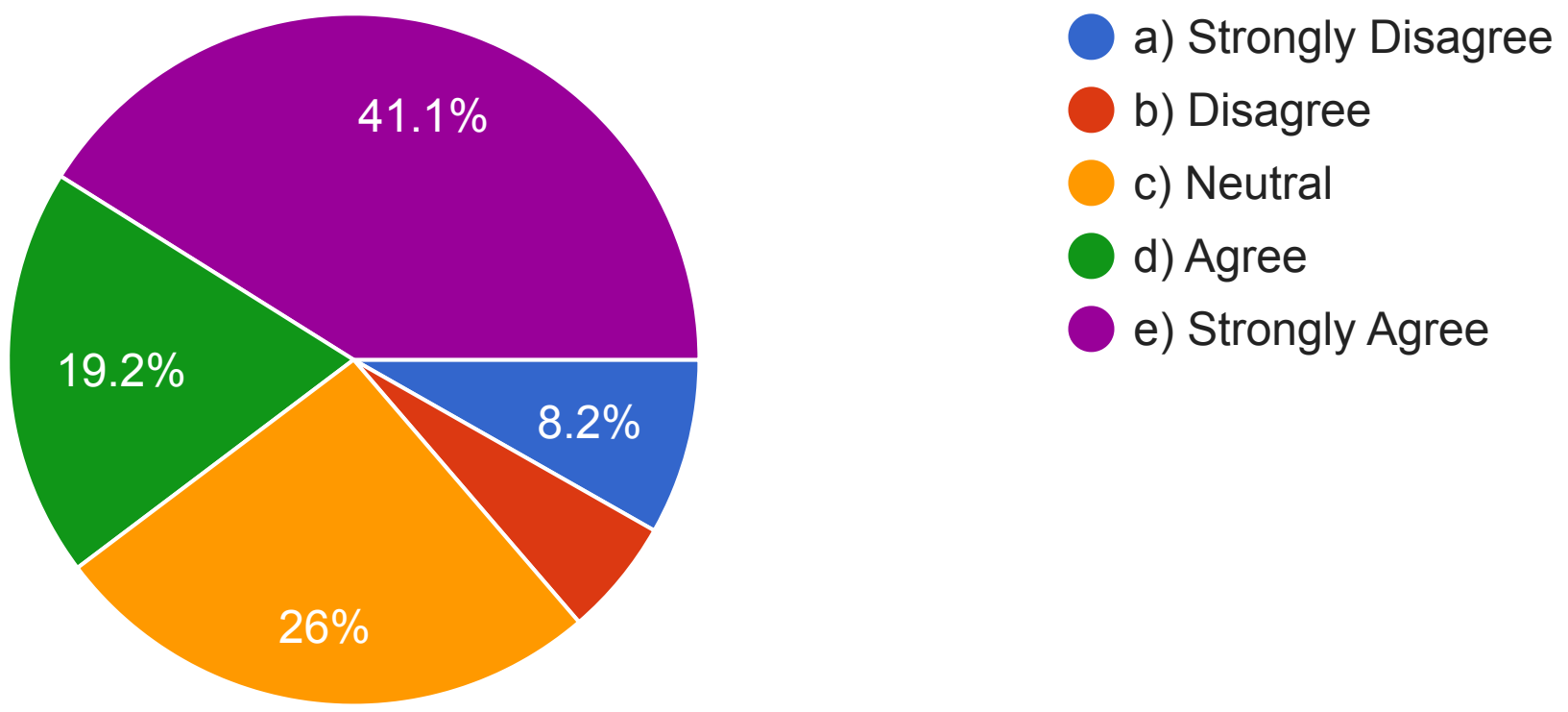

I would be willing to participate in continuing professional development (CPD) or training workshops on forensic odontology.

Copy chart

73 responses

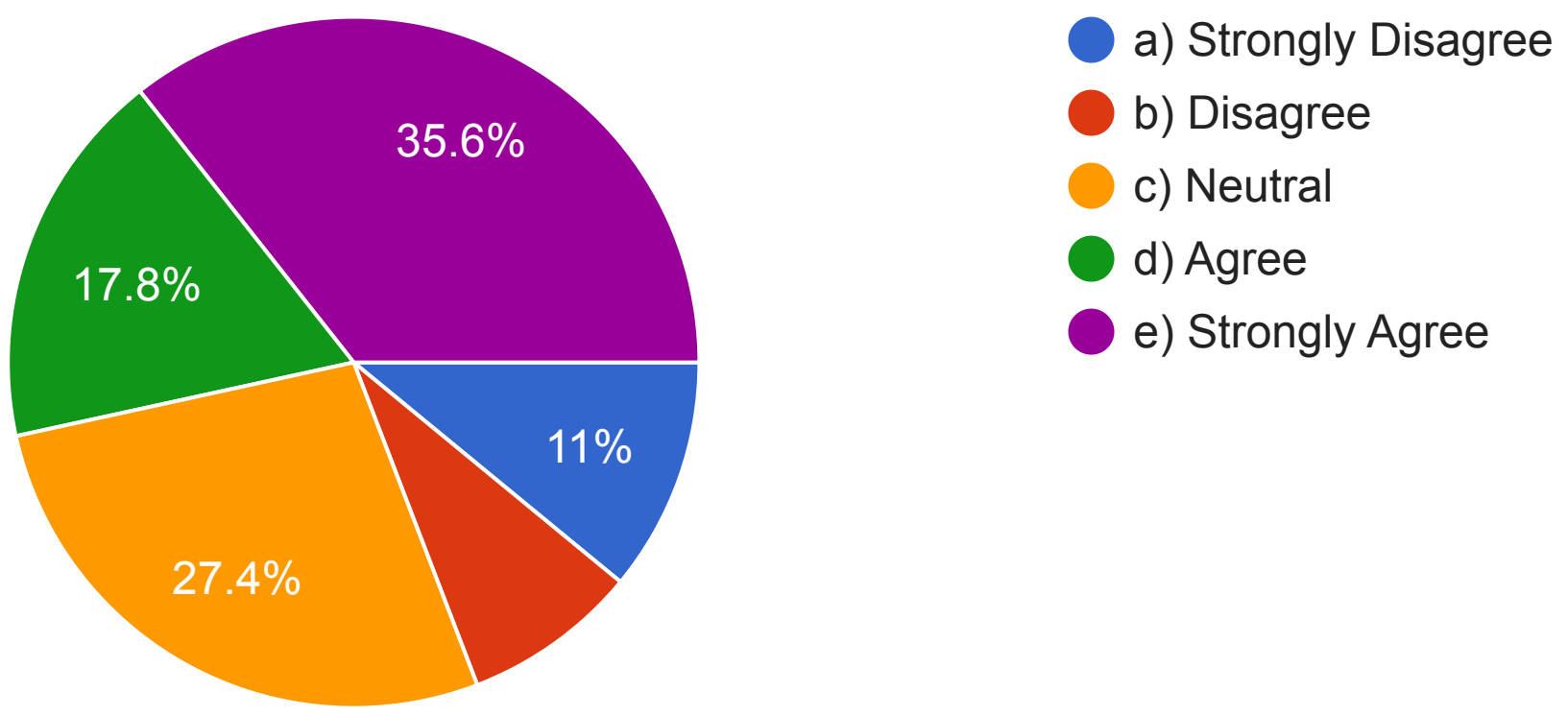

Dentists in Abha and Khamis Mushait currently have adequate knowledge of forensic odontology to meet medico-legal requirements.

Copy chart

73 responses

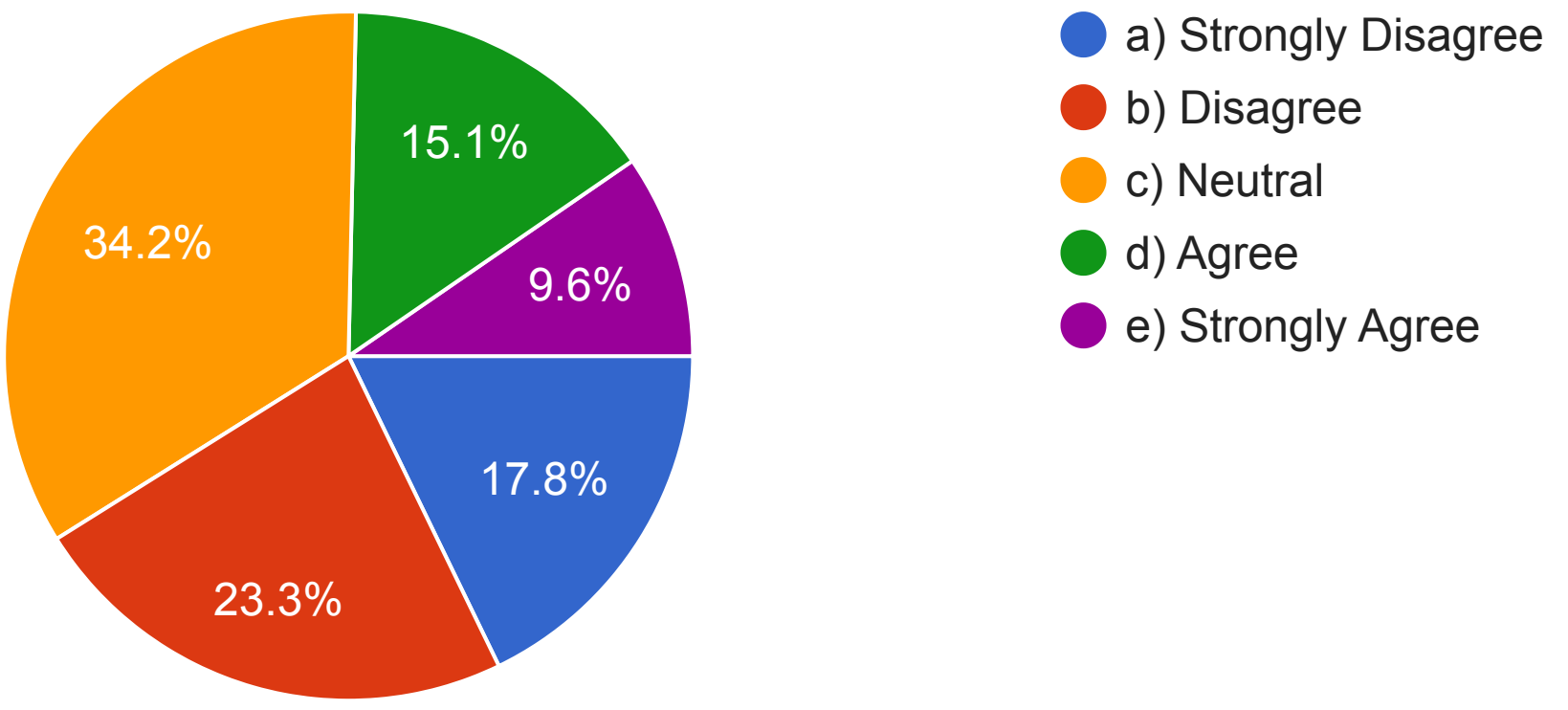

Practice — Dental Record Keeping

Do you routinely document a complete dental history, including past treatments, for all patients?

Copy chart

73 responses

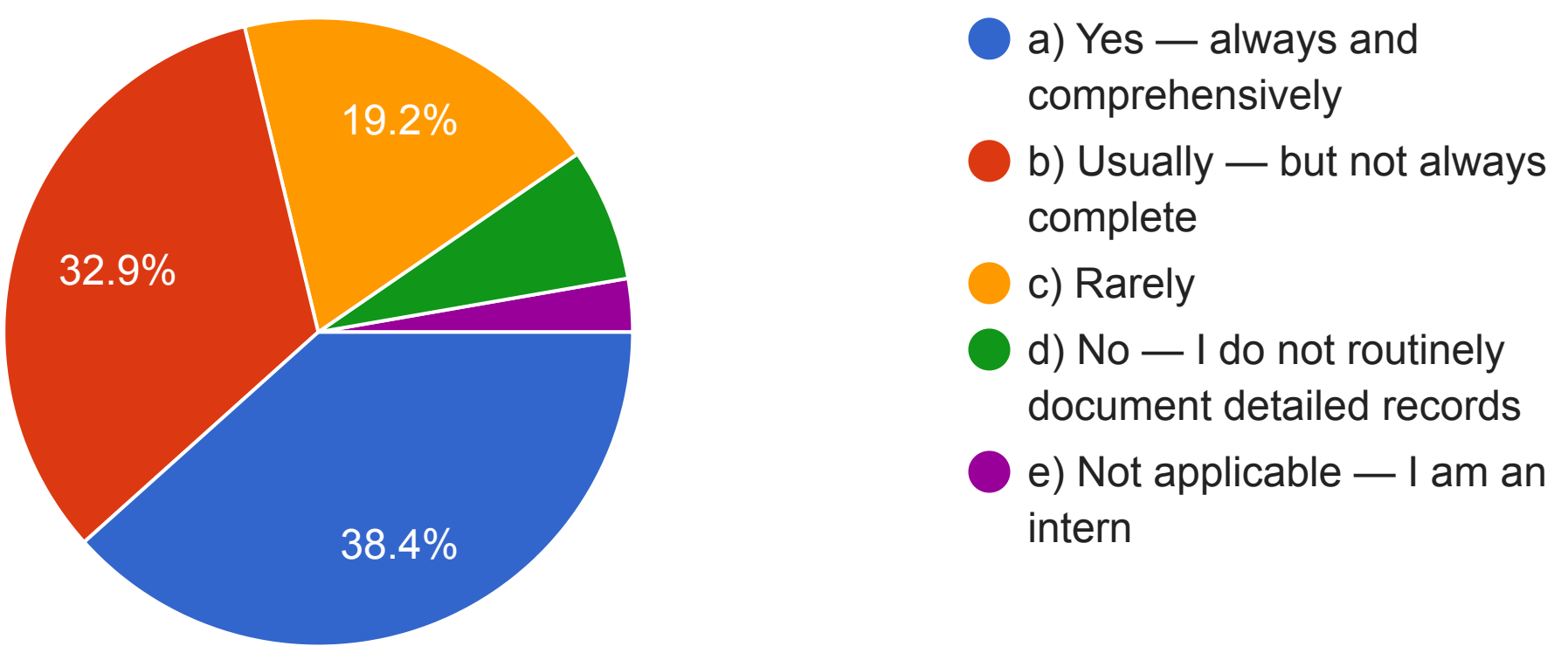

Do you take or prescribe dental radiographs for new patients as a baseline record?

Copy chart

73 responses

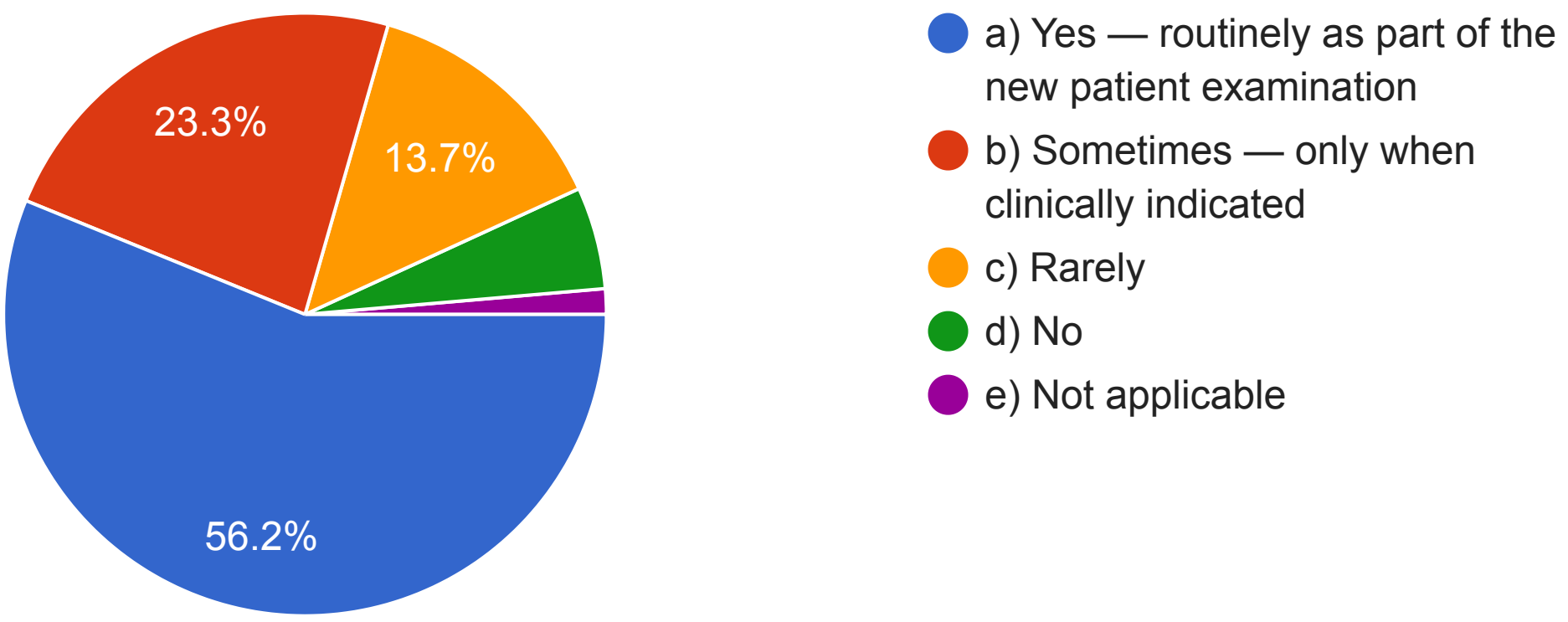

Do you retain and store patient dental records (charts, radiographs, and study models) for at least 10 years?

Copy chart

73 responses

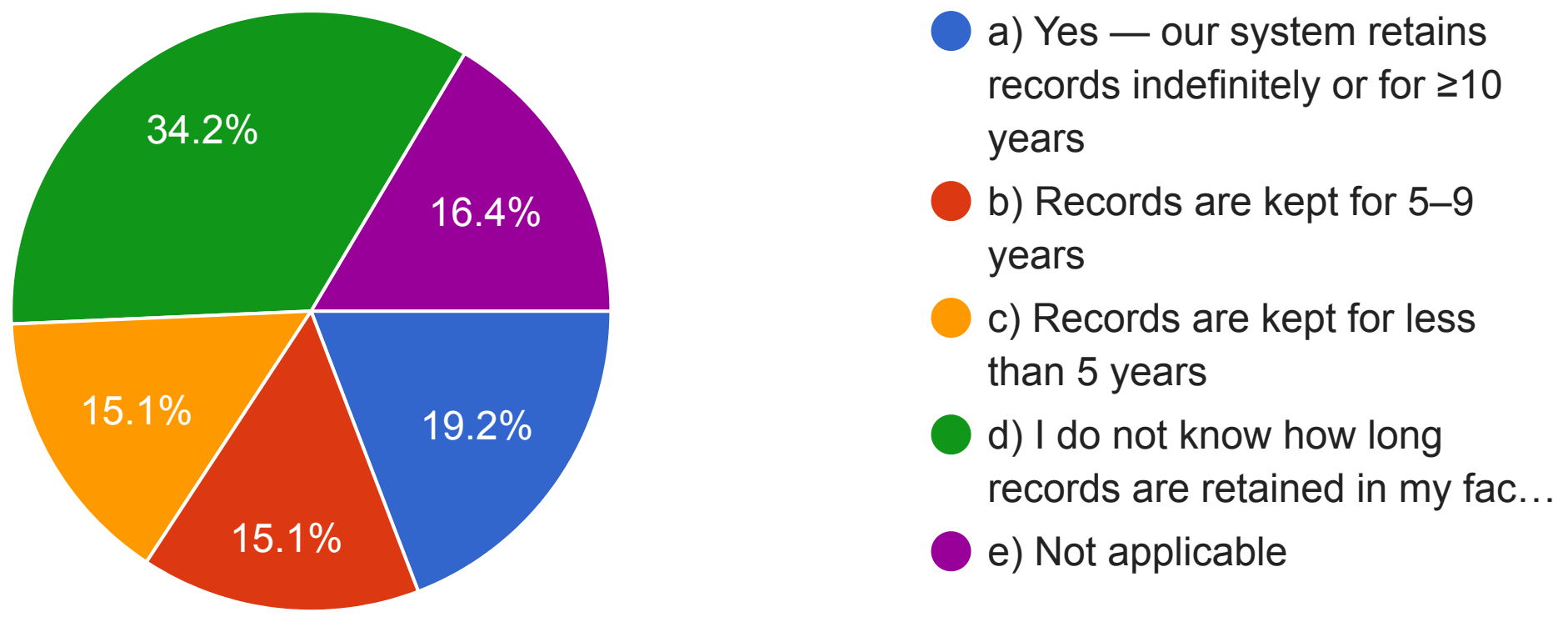

Have you ever encountered a situation in your clinical practice where dental records were requested for medico-legal, forensic, or insurance purposes?

Copy chart

73 responses

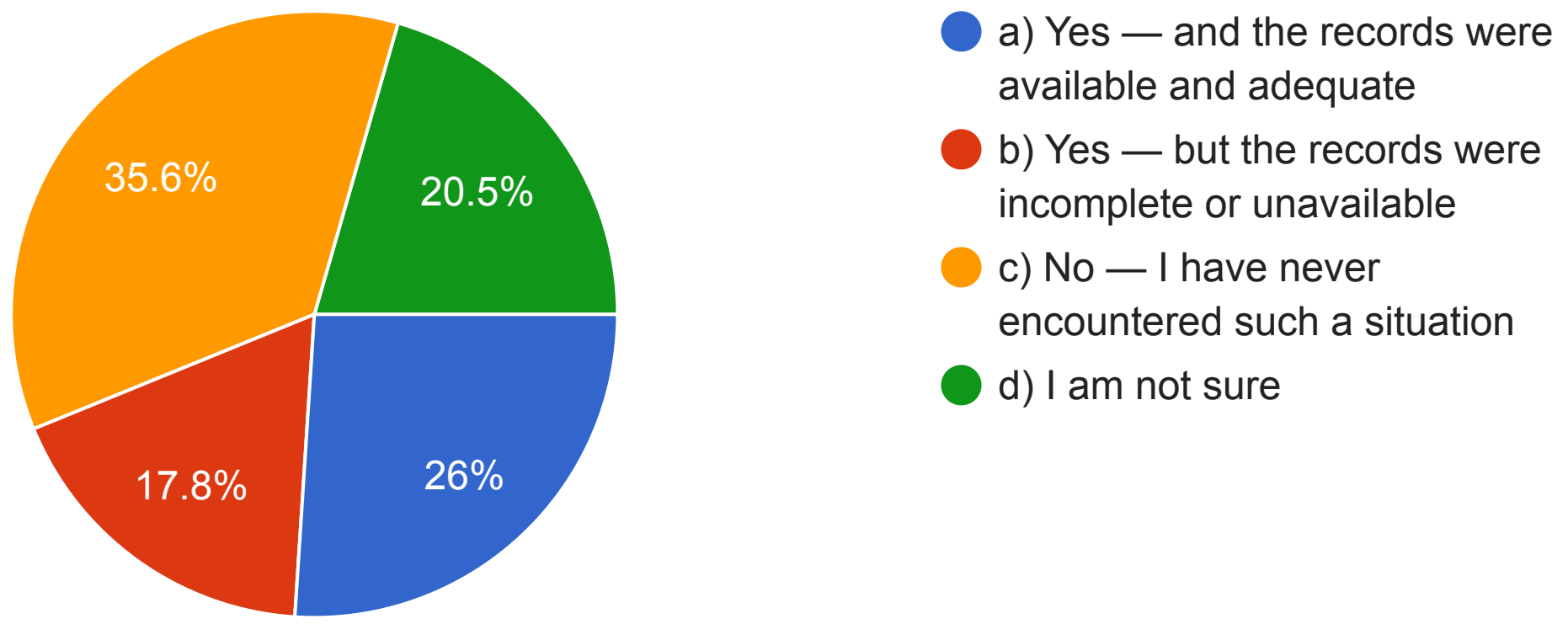

In your opinion, what is the main barrier to adequate dental record keeping in your practice setting? (Select up to TWO)

Copy chart

73 responses

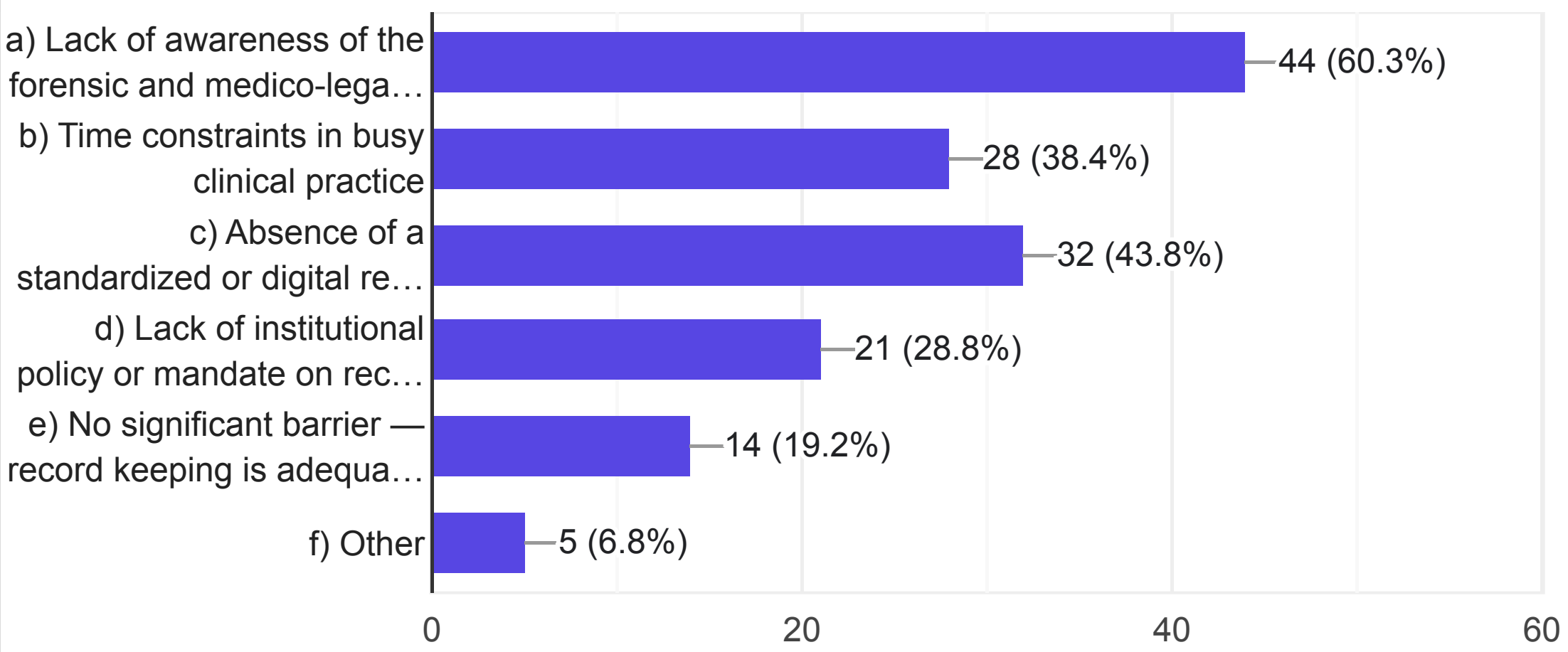

Open-Ended Questions

In your opinion, what specific topics in forensic odontology should be added or strengthened in Saudi dental undergraduate or postgraduate training programs?

73 responses

.

-

..

No

Nothing

all

I Do not know, better that all topic be coverd

Non

Should be an specific postgraduate training program

IDK

A course the talks about every thing in realted to forensic odontology

Case study

data collection and their use in forensic medicine.

ا

The problem wasn't which topic but why they didn't mention this topic at all

I don’t have any idea

Should be add as a subject in undergraduate

We need all the topics and subjects that’ll help us to learn about forensic odontology .

Estimated age

Students need more courses in forensic dentistry

The whole idea of forensic

Forensic odontology training in Saudi dental programs should strengthen dental identification, age estimation, bite mark analysis, DNA handling, forensic imaging, legal knowledge, and disaster victim identification, with a greater focus on hands-on and simulation-based learning.

In my opinion, forensic odontology in Saudi dental programs still needs more focus, especially on practical aspects. Topics like proper dental record keeping should be strongly emphasized, because they play a huge role in patient identification. I also think areas like age estimation, bite mark analysis, and disaster victim identification should be covered in more depth.

Yes

From my perspective.. in undergrad only basic of foresic odontology
And postgrad put Independent path

Lagal

About how to handle a situation like that

In early founding

…

General course and continuous edication courses

my opinion, the following topics should be added to forensic odontology:
• Age estimation techniques using teeth (especially in children and adults)
• Bite mark analysis and its limitations in legal cases
• Dental identification in mass disasters (DMORT protocols)
• DNA extraction from teeth and its forensic applications
• Digital forensics in dentistry (CBCT, AI-based identification)
• Human abuse and neglect recognition through oral findings
• Legal and ethical responsibilities of dentists in forensic cases

Bite marks, lip prints

Add Forensic Course in the plan student

Abuse detection and reporting

Forensic odontology

General forensic odontology

Important of forensic odontology and standard of data and records saved

ن

What is -what is important of forensic

Strengthen forensic odontology training by focusing on dental identification, age estimation, and proper dental record documentation, with more practical and simulatio based learning.

How can we know the age of patient by the the tooth, if we don’t know the date of birth.

Forensic at all

FORENSIC

All

Human Identification Techniques

Knowing forensic odontology in general will be helpful.

Bite mark analysis and dental age estimation.

In genral

To strengthen forensic odontology in Saudi programs, training should prioritize standardized digital record-keeping for victim identification and practical mastery of age estimation methods (like the AlQahtani Atlas). Additionally, curricula must integrate Interpol’s DVI protocols for mass casualty management—essential for Hajj seasons—and provide specialized training on the Saudi judicial system to qualify dentists as expert witnesses in local courts.

I have no idea honestly

Undergraduate

Forensic odontology training should be strengthened by including dental identification, proper dental record keeping, age estimation methods, medico-legal report writing, disaster victim identification, abuse recognition, and the use of modern technologies such as digital imaging and 3D systems.

Nothing

Basic information as first step

No comment

Should be in post in Saudi board should be as a subject during the undergrad

The entire field in overlooked at the undergraduate levels

Cases of violence, especially among children

Do you have any additional comments about forensic odontology education, dental record keeping practices, or the medico-legal role of dentists in the Aseer region?

73 responses

No

.

-

Non

..

No

I hope to find more conference, lectures, workshops about it

I don’t know

No thanks

I believe there should be a committee that consider this field and take responsibility as a reference to go to.

١

.

We need to learn and know more about forensic odontology

Yes we need to improve the knowledge and awareness regarding forensic dentistry

I think there is a need to raise more awareness among dentists in the Aseer region about the importance of proper dental records, as they are essential in forensic and legal cases. Many dentists may not realize how important accurate documentation can be.

Yes

Please open as a diploma or Master's certificate in our region and take all of the benefits of that

IDK

I think we need to consider the learning process for this

…

Increased awareness is required

No thank you

None

no

Need a courses about forensic odontology education

م

Improve awareness of medico-legal responsibilities and standardize dental record keeping practices, with better collaboration between dentists and forensic authorities.

It is good and interesting but very rare to take knowledge about it

No further comments. Thank you

We hope that this specialization will be available as a specialized field after our graduation and linked to the Ministries of Defense and Interior as a dual-purpose operation to facilitate and streamline military and health-related work simultaneously.

In the Aseer region and Saudi Arabia, forensic odontology should be a mandatory part of dental education, with improved dental record keeping practices, a stronger medico-legal role for dentists in identification and reporting, and the establishment of a national dental database to support forensic investigations.

It was my passion to be a forensic doctor after the medical bachelor but I am a dentist when I graduated. I didn't know that if we have a forensic or odontology post

No thank you

Thank you for your participation. Your responses will contribute to advancing forensic odontology education and practice in the Aseer region.

This content is neither created nor endorsed by Google. - Contact form owner - Terms of Service - Privacy Policy

Does this form look suspicious? Report

Google Forms
